# Multi-scale simulations of the T cell receptor reveal its lipid interactions, dynamics and the arrangement of its cytoplasmic region

**DOI:** 10.1371/journal.pcbi.1009232

**Published:** 2021-07-19

**Authors:** Dheeraj Prakaash, Graham P. Cook, Oreste Acuto, Antreas C. Kalli

**Affiliations:** 1 Leeds Institute of Cardiovascular and Metabolic Medicine, School of Medicine, University of Leeds, Leeds, United Kingdom; 2 Astbury Center for Structural Molecular Biology, University of Leeds, Leeds, United Kingdom; 3 Leeds Institute of Medical Research at St James’s, School of Medicine, University of Leeds, Leeds, United Kingdom; 4 Sir William Dunn School of Pathology, University of Oxford, Oxford, United Kingdom; US Army Medical Research and Materiel Command: US Army Medical Research and Development Command, UNITED STATES

## Abstract

The T cell receptor (TCR-CD3) initiates T cell activation by binding to peptides of Major Histocompatibility Complexes (pMHC). The TCR-CD3 topology is well understood but the arrangement and dynamics of its cytoplasmic tails remains unknown, limiting our grasp of the signalling mechanism. Here, we use molecular dynamics simulations and modelling to investigate the entire TCR-CD3 embedded in a model membrane. Our study demonstrates conformational changes in the extracellular and transmembrane domains, and the arrangement of the TCR-CD3 cytoplasmic tails. The cytoplasmic tails formed highly interlaced structures while some tyrosines within the immunoreceptor tyrosine-based activation motifs (ITAMs) penetrated the hydrophobic core of the membrane. Interactions between the cytoplasmic tails and phosphatidylinositol phosphate lipids in the inner membrane leaflet led to the formation of a distinct anionic lipid fingerprint around the TCR-CD3. These results increase our understanding of the TCR-CD3 dynamics and the importance of membrane lipids in regulating T cell activation.

## Author summary

The T cell receptor (TCR-CD3) detects antigenic peptides displayed by major histocompatibility complexes (pMHC) to instigate activation of T cell-mediated adaptive immunity. Despite significant structural and functional knowledge of the TCR-CD3 topology, the membrane interactions and dynamics of its cytoplasmic moieties remain elusive. Interactions of TCR-CD3 cytoplasmic tails with membrane lipids may regulate their phosphorylation by Src-family kinases, the first intracellular event required for T cell activation. Using the static 3D structure of TCR-CD3 resolved by cryo-electron microscopy, we provide novel insights into the protein-lipid interactions of the complete TCR-CD3 embedded in a bilayer closely mimicking its native membrane environment. Our study sheds light on the dynamics of the TCR-CD3 at near-atomic resolution and further aids in deciphering its activation mechanism.

## Introduction

T lymphocytes express a diverse repertoire of antigen receptors known as T cell receptors (TCR-CD3 complexes) on their plasma membrane. TCRs identify peptide antigens displayed in the jaws of major histocompatibility complexes (MHC) [1,2]. The TCR-CD3 complex consists of four non-covalently assembled dimers: TCRαβ, CD3δε, CD3γε heterodimers and the ζζ homodimer [3]. Disulphide bridges aid in linking the subunits within the αβ and ζζ dimers in the extracellular region. Inter-subunit interactions are mediated by the ectodomains (ECDs) and the transmembrane regions (TMRs) which contribute to the stability of the complex and determine its precise topology [3–5]. The α and β subunits each display variable domains, Vα and Vβ, featuring three variable loops of complementarity-determining regions (CDRs) 1, 2 and 3 that together form the VαVβ binding site for peptide-MHC (pMHC) ligands [6]. The cytoplasmic regions (CYRs) of the α and β subunits each contain short peptides of less than ten amino acids long and do not transmit signals to the intracellular region.

TCR-CD3 signal transduction, which is initiated by pMHC binding to TCRαβ, is governed by the phosphorylation of immunoreceptor tyrosine-based activation motifs (ITAMs) in the intracellular region of CD3 and ζ subunits [7]. Circular dichroism (CD) spectroscopy experiments suggested that the CYRs of each ITAM-containing subunit are intrinsically disordered in both monomeric and oligomeric states, but exhibit lipid-binding with acidic phospholipid-containing vesicles [8]. CD3ε and ζ CYRs contain basic-rich stretches (BRS) that have been suggested to mediate robust ionic interactions with negatively charged headgroups of phosphatidylinositols [9,10] and phosphatidylserine [11] in the inner leaflet of the membrane. Moreover, the tyrosine sidechains in the ITAM-containing segments of both CD3ε and ζ CYRs were found to penetrate into the hydrophobic core of the membrane [11,12]. However, it remains unclear whether this configuration applies to all cytoplasmic tyrosines in an entire TCR-CD3 complex. A ‘stand-by’ model of TCR-CD3 signalling was proposed based on the evidence that a pool of constitutively active LCK at the T cell plasma membrane phosphorylates the ITAMs of TCR-CD3 CYRs upon their disengagement from the membrane [13]. However, the molecular mechanism of pMHC binding that induces a change in the ITAM configuration which favours accessibility by LCK remains unclear. The key to these questions potentially lies in the interactions of the TCR-CD3 complex with its local membrane environment which is also currently poorly understood.

A recent cryo-electron microscopy (cryo-EM) study [4] revealed the 3D structure of the human TCR-CD3 complex (PDB:6JXR) at a resolution of 3.7 Å and supports previous findings that TCRαβ maintains critical ionic contacts with CD3δε, CD3γε and ζζ in the TMR [3]. This study shed light on the quaternary structure arrangement featuring highly interlaced contacts among subunits’ ECDs and TMRs, suggesting a dense connectivity maintaining the topology of the entire complex. However, the cryo-EM structure could not identify the arrangement of the CYRs presumably due to their disordered state. Moreover, the dynamic behaviour of the TCR-CD3 ECDs, TMRs and CYRs when embedded in their native membrane environment has not been studied. This information may provide clues to the signal transduction mechanism.

Here, we have used the cryo-EM structure to generate the first molecular model of the entire TCR-CD3 embedded in a complex asymmetric bilayer containing the predominant lipid species found in its native environment upon receptor activation [14]. Our multi-scale molecular dynamics (MD) simulation approach, in coarse-grained and atomistic resolutions, provided insights into the conformational flexibility of the TCR-CD3 and its interactions with membrane lipids in the microseconds timescale. We show that the CYRs assemble into a coiled conformation and interact with the inner membrane leaflet, while anionic headgroups of phosphatidylinositol phosphate (PIP) lipids interact selectively with the TCR-CD3 CYR. Our data also reveal that the ECDs, TMRs, and CYRs each exhibit conformational changes when the TCR-CD3 is embedded within the lipid bilayer, potentially supporting a model of allosteric activation of the TCR-CD3 complex.

## Results

### Modelling the entire TCR-CD3 complex

**Fig 1:**
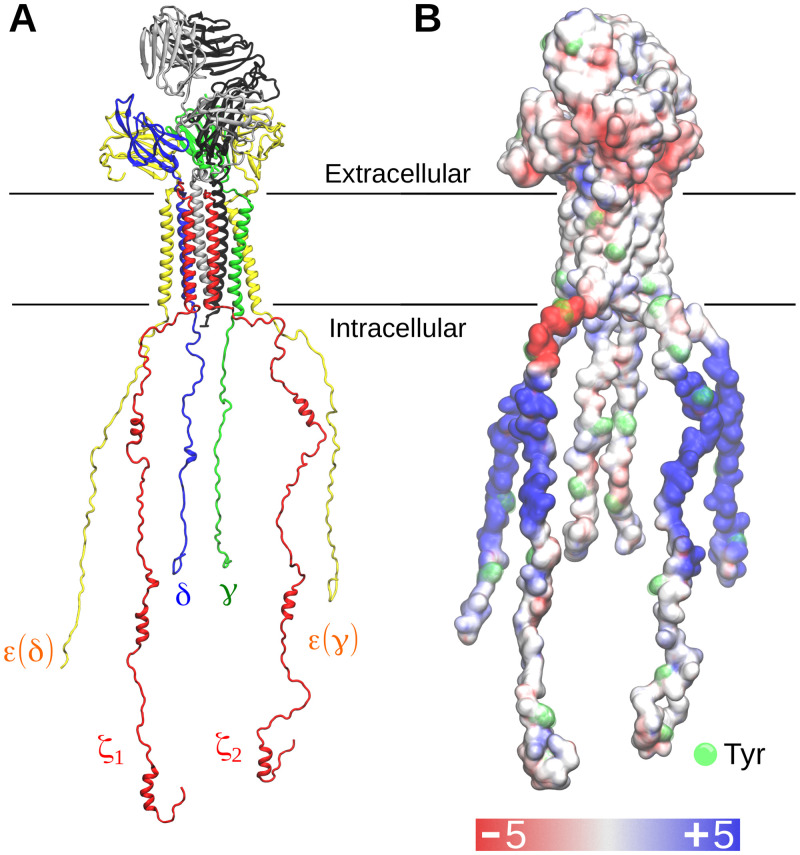
The T cell receptor structure. **(A)** Model of the entire T cell receptor used in our simulations. **(B)** Electrostatic profile of the TCR-CD3. A range of ±5 kT/e was used to indicate the electronegative and electropositive regions as shown in red and blue respectively. The calculation of the electrostatic profile was done using the APBS tool [18].

We used the cryo-EM structure of the human TCR-CD3 complex (PDB:6JXR) as a template and performed secondary structure predictions with the PSIPRED 4.0 workbench [15] using sequence data from Uniprot (see Methods) to model the entire complex. The predictions suggested that the cytoplasmic region (CYR) of the CD3δ, γ, ε subunits lacked secondary structure in agreement with circular dichroism experiments [8]. However, the ζ CYR was predicted to contain short α-helices consistent with NMR spectroscopy data [16]. These regions were modelled as helical in our complete TCR-CD3 structure while the rest of the ζ CYR were modelled as unstructured regions. Some extracellular residues in the CD3ε, CD3γ, TCRα subunits that were missing from the cryo-EM structure were also modelled as unstructured regions (S1A Fig). The CYRs of the CD3 and ζ subunits were modelled in a linear extended orientation perpendicular to the membrane to avoid bias in inter-subunit contacts at the beginning of the simulations. During our modelling, the TMRs and the ECDs of the TCR-CD3 subunits were position-restrained to preserve their experimentally derived structural integrity. Finally, twenty different models including each of their discrete optimized protein energy (DOPE) scores [17] were obtained. The structural models showed minimal differences from one another due to position restraints applied on the tertiary structure of the ECDs and TMRs, and on the predicted α-helices in both the ζ CYRs. The most energetically favourable model (least DOPE score) of the entire TCR-CD3 complex was used and further energy minimized. This atomistic model (Fig 1) was converted to coarse-grained (CG) resolution using the Martini 2.2 forcefield and used for CGMD simulations.

### The TCR-CD3 cytoplasmic region exhibits a coiled conformation

**Fig 2:**
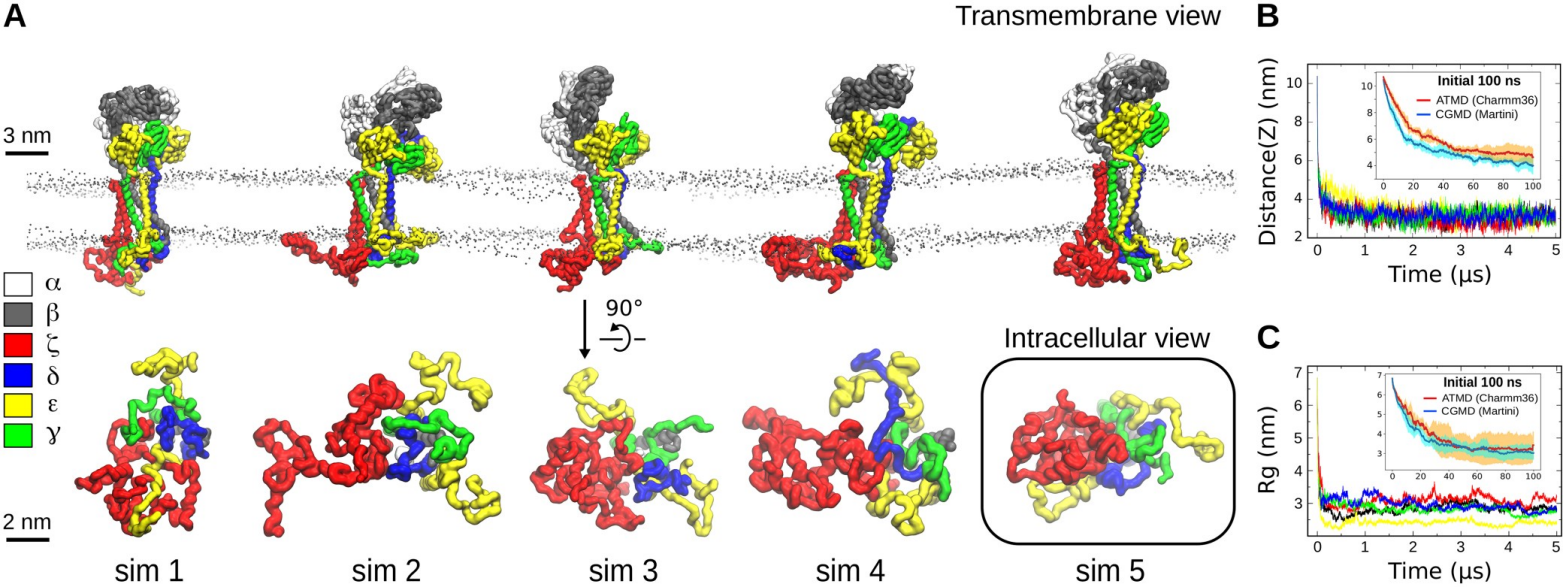
Association of the TCR-CD3 cytoplasmic region with the membrane. **(A)** Side view of the entire TCR-CD3 (top) and intracellular view of its cytoplasmic region (bottom) taken from the end of the five CGMD simulations of membrane 1. The box highlights the most common CYR conformation calculated by the clustering analysis. **(B)** Distances between the center of mass (COM) of the TCR-CD3 CYR and the COM of membrane 1 calculated from the five CGMD simulations along the vertical (Z) axis versus time. **(C)** Radius of gyration (Rg) of the TCR-CD3 CYR versus time from all CGMD simulations. In (B) and (C), distance(Z) and Rg were also calculated from ATMD simulations performed for 100 ns. Comparison of the average distance(Z) and Rg between the ATMD (red) and CGMD (blue) simulations is shown in the inset graph. Standard deviation is shown in cyan for CGMD and in orange for ATMD.

The CGMD simulations of the entire TCR-CD3 complex were used to analyse its dynamic nature when embedded in a lipid bilayer comprised of the predominant lipid types found in the TCR-CD3 activation domain (Table 1) [14]. Five independent CGMD simulations of the TCR-CD3 were performed in an asymmetric complex membrane for 5 μs each. During the simulations, the CYRs of ζζ and CD3 dimers that were initially modelled in an extended configuration, rapidly coiled forming inter-chain interactions and then associated with membrane lipids of the inner leaflet (Fig 2A). Calculation of the distance between the center of mass (COM) of the CYRs and the COM of the lipid bilayer as a function of time suggested that the association of the CYRs with the membrane occurred within the first 100 ns of the simulations. Although the time taken for the CYRs to coil and associate with the membrane was consistent amongst all CGMD simulations, their radius of gyration varied indicating that the coiled conformation of the CYRs is dynamic. To confirm the propensity of the CYR of TCR-CD3 to form coiled structures and to associate with the membrane, we have also performed ATMD simulations for 100 ns starting from the same initial model (cytoplasmic tails were in an extended conformation perpendicular to the membrane). These simulations confirmed our previous observation that TCR-CD3 CYR can form coiled structures and associate with the membrane (Fig 2B and 2C and S1B Fig). Due to the fluctuation in the CYR assembly, we wanted to determine its most commonly occurring structural conformation. Therefore, using the coiled and membrane-bound state of the CYRs (from the last 4 μs of the CGMD simulations), we extracted 10,000 cytoplasmic configurations from all simulations combined and grouped them into clusters using a 3.5 Å RMSD cut-off (RMSD calculated relative to our initial model shown in Fig 1). The largest cluster contained 867 similar structures (S1C Fig) representing the most frequent structural conformation of the CYRs across all simulations (shown in a box in Fig 2A).

**Table 1:** Composition of lipid headgroups (%) in each leaflet of the membrane in all simulations.

Lipid concentration (%)	PC	PE	SM	CHOL	PS	PIP_2_	PIP_3_
**Outer leaflet**	50	10	20	20	-	-	-
**Inner leaflet**	10	40	-	20	20	8	2

### Membrane penetration by ITAM tyrosines

**Fig 3:**
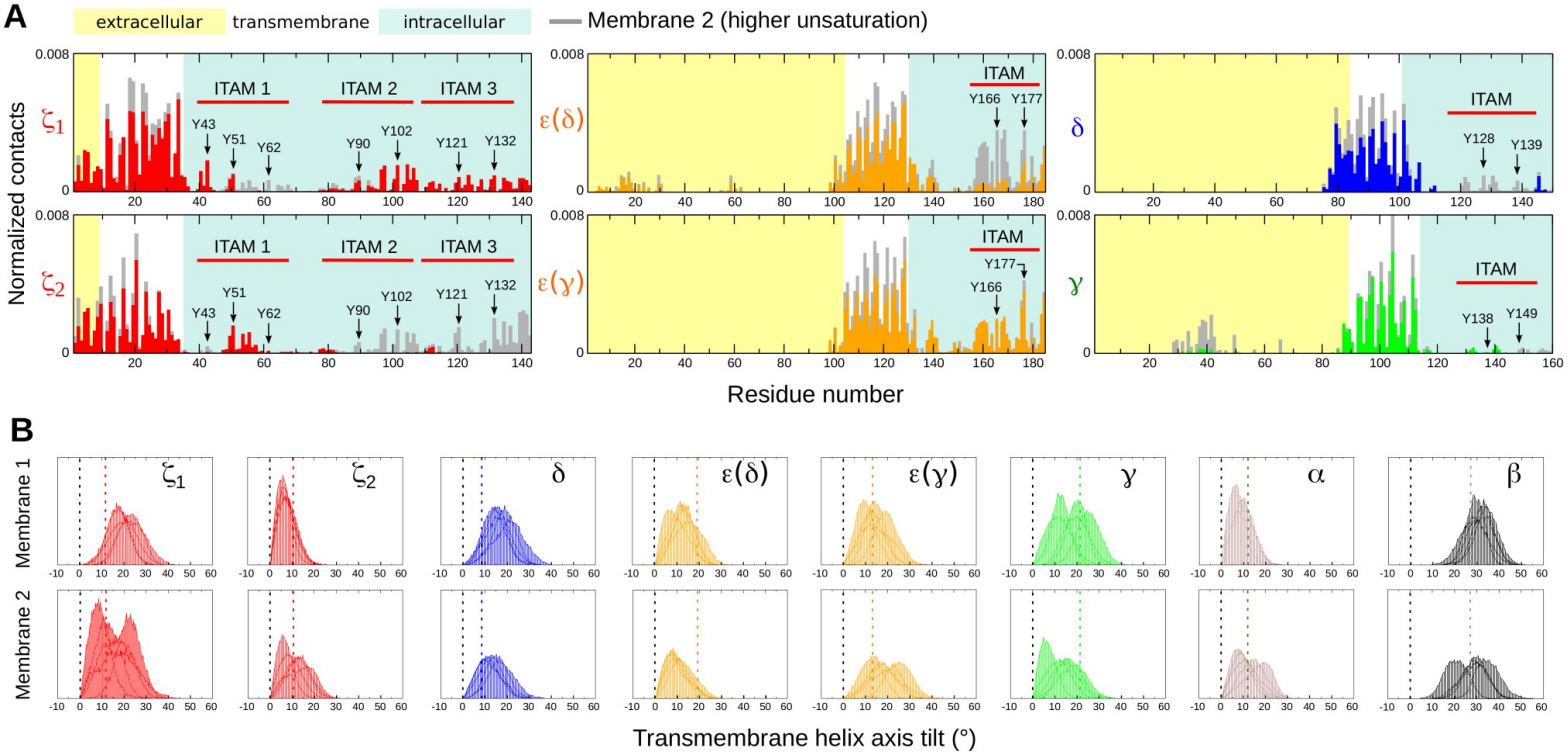
Interactions of ITAM tyrosines with the hydrophobic region of the membrane. **(A)** Normalized number of contacts of ζ and CD3 subunits with lipid acyl chains in membrane 1 (coloured by subunit) and membrane 2 (grey). The position of the ITAM tyrosines is indicated by arrows. For the normalization, the number of contacts of each residue was divided by the number of lipids in the membrane and the number of simulation frames. **(B)** Transmembrane helix tilt angle distribution of each subunit calculated from all simulations from membrane 1 (top row) and membrane 2 (bottom row). The black dotted lines represent zero tilt angle and the coloured dotted lines represent the initial tilt angles of the transmembrane helices of each subunit.

Previous NMR studies of the CD3ε ITAM-containing peptide interacting with a lipid micelle suggested that two ITAM tyrosines, one isoleucine and one leucine residue penetrated the hydrophobic core of the membrane [11]. To investigate whether our simulations showed similar membrane-penetrating activity of the ITAM tyrosines, we calculated their interactions with the hydrophobic acyl chains of the lipids in our CGMD simulations. We found that membrane penetration was only achieved by some ITAM tyrosines. In all simulations combined, the tyrosines that displayed the most membrane-penetrating capabilities belonged to the CD3ε and ζ subunits only. Y177 of CD3ε pairing with γ, referred to as CD3ε(γ), made the highest number of contacts with the lipid acyl chains (S1 Movie), followed by Y177 of CD3ε(δ). Y166 of CD3ε(γ) penetrated the membrane more than Y166 of CD3ε(δ). In addition, only one of the subunits of the ζζ dimer mostly showed ITAM tyrosine contacts with lipid acyl chains. We also calculated the contacts of the entire TCR-CD3 subunits with the lipid acyl chains (Fig 3A). Interestingly, the short extracellular segments of both ζ subunits contacted the acyl chains of lipids in the outer membrane leaflet suggesting their tendency to anchor onto the extracellular leaflet during the simulations. In these simulations, we have used mono-unsaturated phospholipids. Given our finding that tyrosine residues on ITAMs penetrate the membrane, it is possible that a higher unsaturation of the phospholipid acyl chains may facilitate tyrosine penetration even further. Therefore, as a control, we conducted simulations in which the TCR-CD3 was inserted in a bilayer containing the same concentration of lipid headgroups but the degree of lipid tail unsaturation was increased. The new membrane composition is shown in S2A Fig. The increase in unsaturation resulted in similar tyrosine penetration with a somewhat higher penetration of some tyrosines of the ζ2 and the ε(δ) CYRs (Fig 3A). This observation augments our previous observation that tyrosine residues are able to penetrate the bilayer when the TCR-CD3 is in a steady state. We also calculated the change in TMR tilt angle for each of the subunits to assess the influence of membrane unsaturation and observed that the range of tilt angles for all TMRs were similar but with minor differences in the peak of the distribution of the tilt angles (Fig 3B).

### TCR-CD3 selectively interacts with lipid headgroups

**Fig 4:**
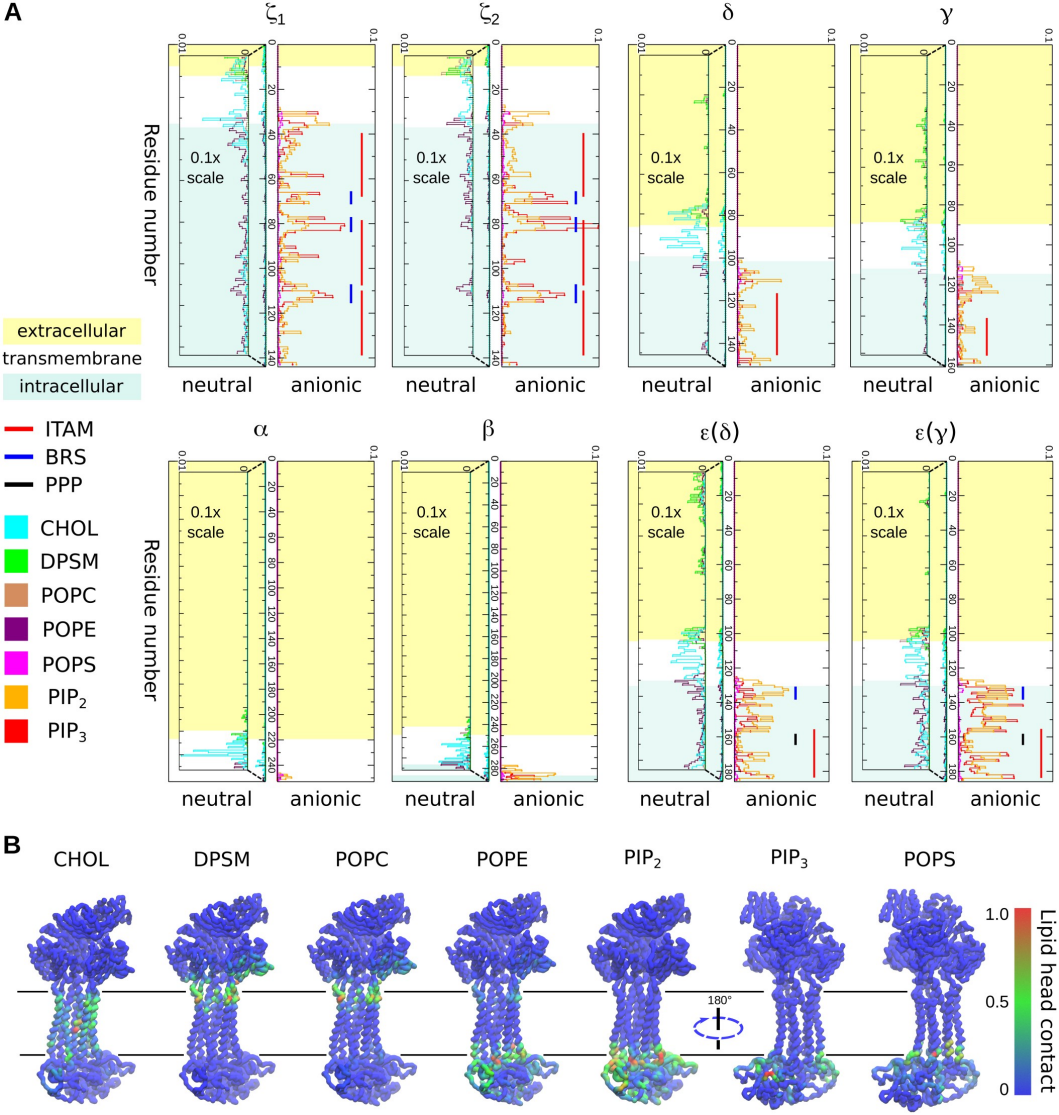
Interactions of the TCR-CD3 with lipid headgroups. **(A)** Normalized number of contacts of each full-length TCR-CD3 subunit with lipid headgroups. The normalization (N) is done by dividing the number of lipid contacts of each residue (n1) by the number of the specific lipid in the bilayer (n2) and by the number of simulation frames (n3) i.e. N = n1/n2/n3. Interactions of each TCR-CD3 subunit with anionic lipids are shown on the right whereas those with neutral lipids are shown on the left. The scale of non-anionic interactions was magnified 10 times (scale: 0 to 0.1) for clarity. The ITAMs (red lines) of ζ and all CD3 subunits, the BRS motifs (blue lines) of CD3ε and ζ subunits, and poly-proline motifs (black lines) of the CD3ε subunits are also indicated. **(B)** Contacts of the TCR-CD3 with each lipid headgroup type are normalized separately on a scale of 0 to 1 and mapped as a colour gradient (blue: low, green: medium, red: high) on the TCR-CD3 structure extracted from the end of a simulation.

We questioned if the association of the CYRs onto the inner leaflet of the bilayer affected the TCR-CD3 lipid environment. From all CGMD simulations combined, we analysed the contacts of the entire TCR-CD3 complex with all lipid headgroups including the sterol in both leaflets of the membrane, and further normalized the number of interactions of each lipid-type by their respective concentrations in membrane 1. These data showed the relative enrichment of certain lipids over others in the vicinity of the TCR-CD3 complex. The TCR-CD3 showed a high propensity to contact phosphatidylinositol 4,5-biphosphate (PIP_2_) and phosphatidylinositol 3,4,5-triphosphate (PIP_3_) (Fig 4A and 4B) despite their relatively low abundance in the inner leaflet (8% and 2% respectively). A closer inspection revealed that the cationic residues in the CYRs of CD3ε and ζ subunits dominated the interaction with PIP_2_ and PIP_3_ lipids. The CD3δ and CD3γ CYRs also contacted the PIP lipid headgroups, though to a lower extent. In the CD3ε and ζ subunits, the basic residue-rich stretches (BRS) interacted most with PIPs, while the poly-proline motifs (PPPVPNP) in both CD3ε subunits (labelled ‘PPP’ in Fig 4A) showed the least contact. Basic residues at the TMR-CYR interface of the ζ subunits (R31, K33, R36) also contacted PIP lipids. Other anionic lipid headgroups i.e. POPS, and also neutral lipids made contacts with the CD3 and ζ CYRs but less frequently when compared with PIP lipids (Fig 4A and 4B).

Cholesterol interactions occurred across all the TMRs of the TCR-CD3, and were accompanied by minor interactions with CYRs of CD3ε and ζ (Fig 4A), explained by their ability to penetrate the membrane’s surface. POPE headgroups in the inner membrane leaflet also interacted with the CYRs of CD3ε and ζ to an extent similar to cholesterol interactions (Fig 4A and 4B). Sphingomyelin (DPSM), present only in the outer leaflet, interacted with all subunits but mostly contacted the extracellular segment of ζ and the ECD of CD3ε(δ), followed by the other CD3 subunits. We also observed that POPC headgroups in the outer leaflet interacted with the extracellular segment of ζ and with the connecting peptides (CPs) of CD3 subunits (Fig 4A and 4B). While in agreement with previous studies suggesting ionic interactions of CYRs with the plasma membrane [19], our data points to a potentially more complex scenario of contacts of the TCR-CD3 with membrane lipid headgroups than previously suggested.

### PIP clustering and the significance of the cytoplasmic region

**Fig 5:**
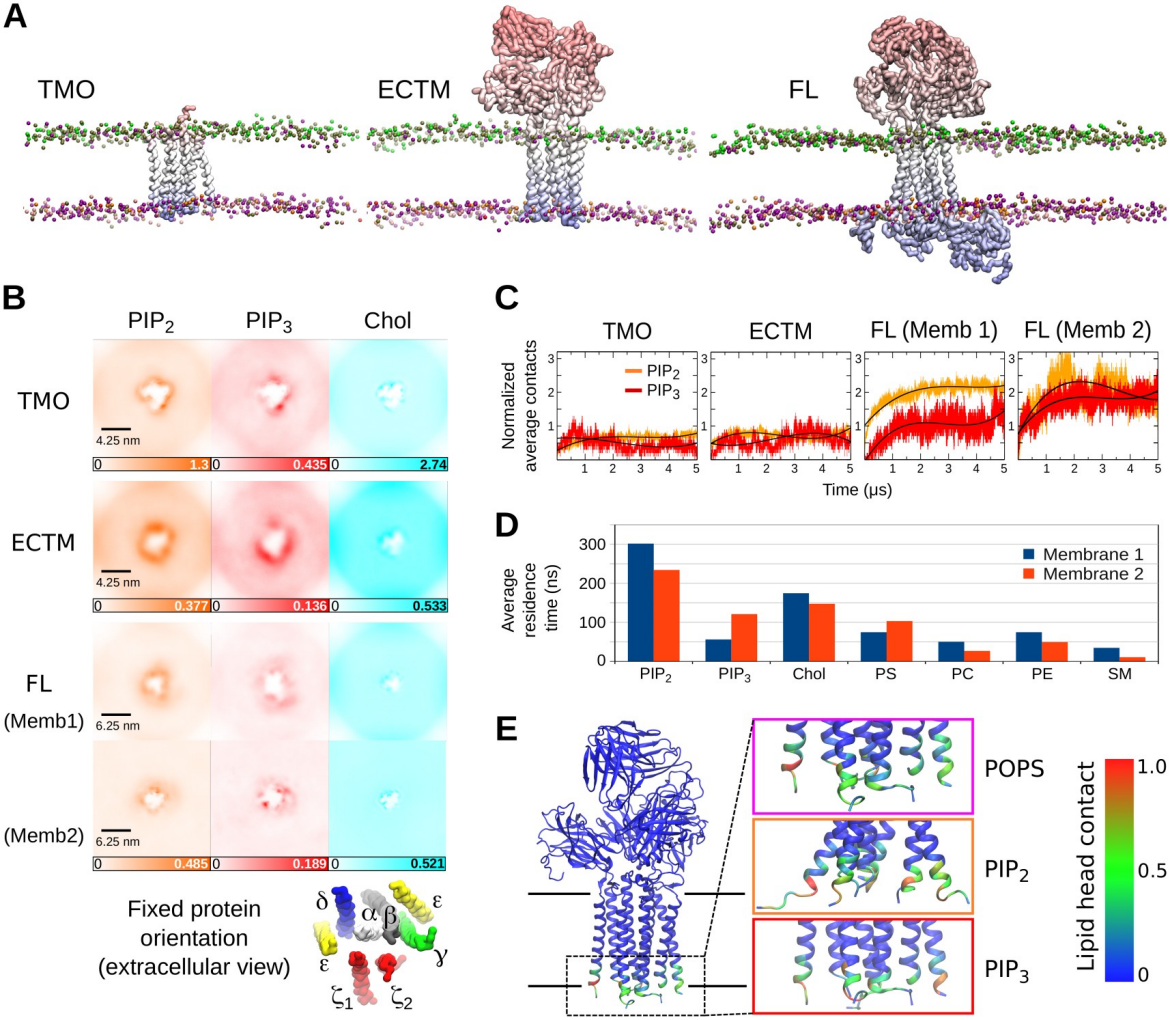
Cholesterol interactions and clustering of PIP lipids around the TCR-CD3. **(A)** Snapshots from the three sets of CGMD simulations (TMO, ECTM, FL) performed in this study. Here, only the protein and the lipid phosphate groups are shown for clarity. **(B)** Extracellular view of the densities of PIP_2_, PIP_3_ and cholesterol molecules in the XY plane of the membrane from all five CGMD simulations combined. The protein was fixed in the center and its TMR orientation is shown below. **(C)** Normalized average number of PIP_2_ and PIP_3_ lipids contacting the protein in all CGMD simulations (TMO, ECTM, FL) versus time. Normalization was done by dividing the number of PIP_2_ and PIP_3_ lipid contacts by their respective number in the membrane. The smoothened black lines are a cubic regression of the number of PIP contacts across time. **(D)** Average residence time of all lipid types in membrane 1 and 2. **(E)** Interaction of the TCR-CD3 cationic anchor with anionic lipids mapped onto the ECTM structure.

Our investigation of PIP interactions with TCR-CD3 CYRs suggested clustering of PIPs around the TCR-CD3 complex. This led us to analyse the densities of each lipid type around the TCR-CD3. In the outer leaflet, there was no clustering of POPE, POPC, and DPSM, but in the inner leaflet the densities of PIP lipids around the protein dominated that of other lipids. To better discern the origin of this effect, we quantified the contribution of the ECDs, TMRs and CYRs of the TCR-CD3 towards lipid interaction. In addition to the CGMD simulations of the full TCR-CD3 complex (referred herein as FL), we performed two sets of CGMD simulations (5 simulations x 5 μs each) using the lipid composition of membrane 1: (1) we excluded the ECDs and CYRs, and retained only the TMRs (referred herein as TMO), (2) we excluded only the CYRs, and retained the ECDs and TMRs (referred herein as ECTM) (Fig 5A). S2B Fig shows the protein sequences used for ECTM and TMO. We calculated the densities of lipids combining all five repeats of the TMO and ECTM simulations and compared them to the densities retrieved from FL simulations. The most striking observation in all three conditions was the clustering of PIP lipids. To assess the influence of poly-unsaturated lipid acyl chains on lipid clustering, we compared the densities of lipids around TCR-FL in membrane 2 to the densities around TCR-FL in membrane 1 and found that they were similar (Fig 5B and S2C Fig). We also calculated the radial distribution functions of all lipid types in membrane 1 and 2, and observed that PIP lipids interacted the most with the TCR-CD3 irrespective of the difference in lipid unsaturation level (S2D Fig).

A distinct cholesterol annulus was clearly observed in the TMO simulations concentrated on the opposite side of the ζζ dimer, suggesting it to be a cholesterol-binding hotspot. Cholesterol bound to similar sites in the ECTM and FL simulations was also observed (Fig 5B). We also found that the average number of cholesterol molecules interacting with the TCR-CD3 were consistent in all three conditions (S2E Fig), suggesting that cholesterol binds to the TMRs independent of the ECDs or CYRs (S3A Fig). Similarly, we quantified the change in the average number of PIP lipids contacting the TCR-CD3 across time. This revealed a three-fold increase in the number of one or both PIP_2_/PIP_3_ lipids in the FL simulations compared to the TMO and ECTM simulations (Fig 5C), suggesting that the CYRs played a significant role in enhancing PIP clustering. The number of interacting POPE lipids with the TCR-CD3 also increased in presence of the CYRs while the number of interacting POPS lipids showed a minor increase. There were no differences in the average number of interacting POPC and DPSM lipids (S2E Fig) when comparing the FL simulations to TMO and ECTM simulations. Calculation of the lipid residence times shows that on average, PIP_2_ lipids spent the most amount of time bound to the protein without detaching followed by cholesterol, PIP_3_ and PS, and other lipids. This order was maintained in membrane 2 where the unsaturation of lipid acyl chains in the inner leaflet was higher than that of the inner leaflet in membrane 1 (Fig 5D and S3B Fig).

We further questioned why there were some anionic lipid headgroups contacting the TCR-CD3 in the ECTM simulations despite the absence of the CYRs. This was due to the cationic residues present at the juxtamembrane region of TCR-CD3 (Fig 5E and S3C Fig), collectively termed here as the ‘cationic anchor’. A multiple sequence alignment of the TMRs and the juxtamembrane region indicates that the cationic anchor is conserved across various species (S3D Fig). Consistently, our analysis suggested that the binding sites of PIP_2_, PIP_3_, and POPS (S4A, S4B and S4C Fig) in the TMO and ECTM simulations occur largely in the juxtamembrane region of CD3ε and ζ subunits. In the FL simulations, binding of anionic headgroups occurred both in their juxtamembrane regions and CYRs. In comparison, the interaction of the TCR-CD3 with anionic lipid headgroups in the absence of its CYRs potentially suggest that the cationic anchor can alone retain approximately one-third of anionic lipids around the TCR-CD3.

### Conformational changes and inter-chain interactions in the TCR-CD3

**Fig 6:**
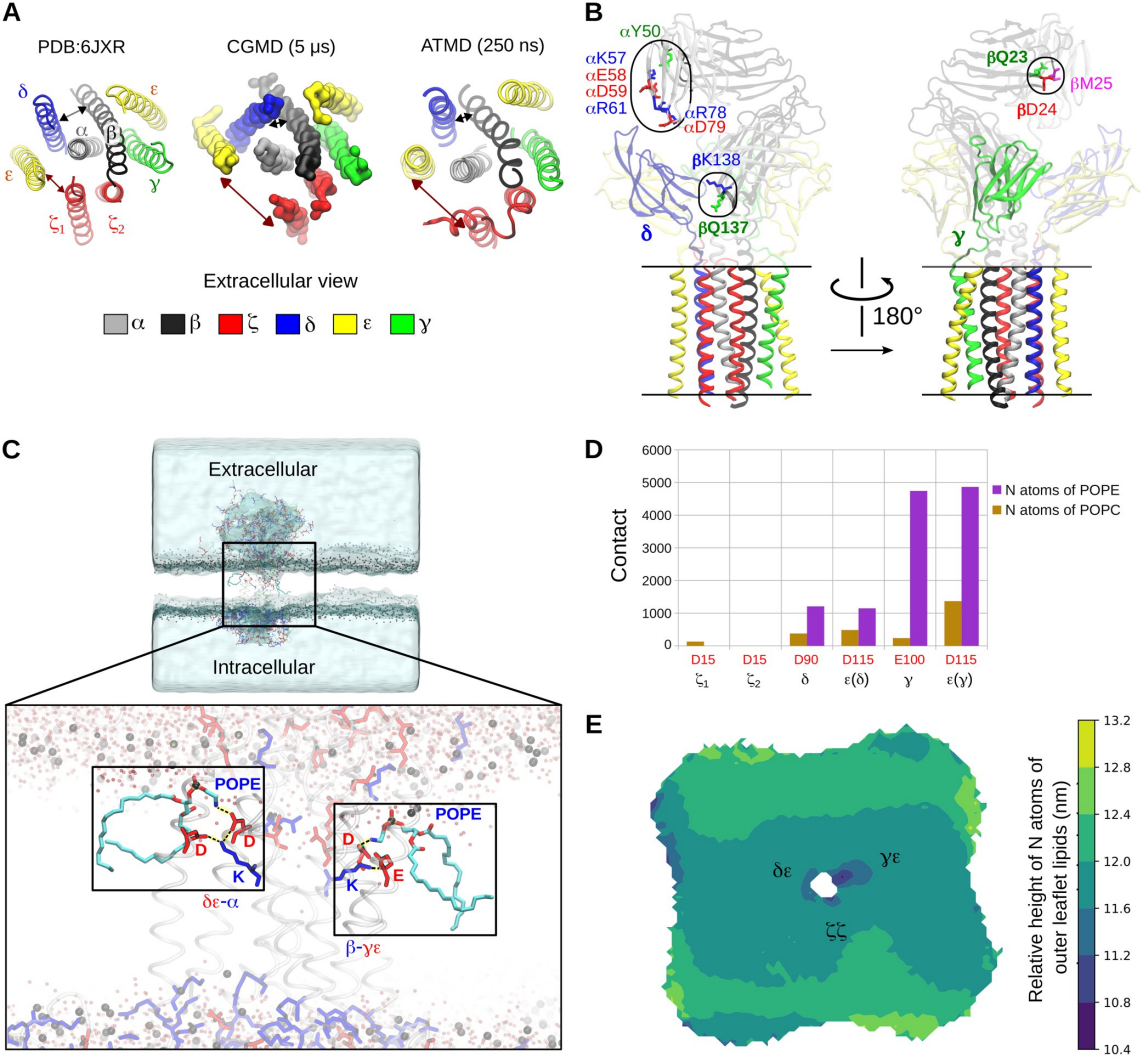
Conformational changes within the TCR-CD3 during our simulations. **(A)** Extracellular view of the TCR-CD3 TMR conformation from the cryo-EM structure (PDB:6JXR) compared to the TMR conformational change seen in the CGMD simulations. This conformational change was retained in the backmapped ATMD simulations throughout the 250 ns simulation time. **(B)** New interactions of the TCRα variable domain and of TCRβ constant domain with CD3δ (left), and of the TCRβ variable domain with CD3γ (right) seen in the backmapped ATMD simulations. **(C)** Snapshot from the end of one of our backmapped ATMD simulations of the entire TCR-CD3. The regions highlighted in boxes show pulled-in POPE headgroups whose amine (NH3) groups interact with the anionic sidechains of CD3 TMRs. The pulled-in POPE lipids are shown in cyan with their oxygen atoms shown in red and nitrogen atoms in blue. Hydrogen atoms are not shown for clarity. Anionic and cationic protein residues are represented as licorice sticks in red and blue, respectively. The TMR helices are shown as transparent cartoons. Water molecules are shown as transparent red spheres and the phosphorus atoms of all phospholipids are shown as black spheres defining the surface of the membrane. **(D)** Number of contacts of anionic residues of CD3 TMRs with nitrogen (N) atoms of POPC and POPE lipids from all backmapped ATMD simulations combined. **(E)** Extracellular view of the relative height of nitrogen atoms of all outer leaflet lipids (POPC, POPE, DPSM) mapped as a colour gradient from dark blue (low) to yellow (high). The TCR-CD3 TMR orientation is fixed in the center as shown in (A).

To determine the conformational changes occurring within the ECD, TMR, and CYRs of the complete TCR-CD3, we first calculated the backbone RMSD of each of these regions. Calculation of the RMSD of the ECD backbone during the simulations confirmed its flexibility and also indicated some distinct conformations (S5A Fig). We also calculated the backbone RMSF of all residues in the complete TCR-CD3 and mapped the values onto the structure highlighting the large fluctuations in the CYRs due to their dynamics (S5B Fig). Inspection of the TMRs in the TMO, ECTM, and FL simulations at the end of 5 μs revealed a consistent loosening of the ζ1 TMR from the CD3ε(δ) TMR (Fig 6A and S5C Fig). Note that, in the cryo-EM structure (PDB:6JXR), their TMRs are in contact only at their N-terminal ends near the surface of the outer membrane leaflet. Calculation of the distance between the COM of ζ1 and of CD3ε(δ) TMRs in the FL simulations showed an increase in the distance between their TMRs (along the XY plane i.e. parallel to the membrane) compared to their initial distance calculated from the cryo-EM structure in 7 out of 10 simulations (S5D Fig). In addition, we observed a decrease in the distance between CD3δ and TCRβ TMRs in 9 out of 10 FL simulations (S5E Fig). During the simulations, the ζζ CYRs also formed contacts with CD3 CYRs (S6A Fig). TCRαβ-ζζ interactions occurred mostly in the TMR with some interactions observed in the membrane-proximal extracellular region. In S6B Fig, we show the interactions of TCRαβ with CD3δε and CD3γε highlighting the contacts of the DE loop in the TCRα constant domain and FG loop in the TCRβ constant domain.

Although the cryo-EM structure shows that the CD3 ECDs are only contacted by the TCRαβ constant domains, our CGMD simulations revealed additional interactions of the TCRαβ variable domain with the CD3 ECDs (S6B Fig) indicating some flexibility within the TCR-CD3 ECDs (shown in Fig 2A **top panel**). To analyse the flexibility of each TCR-CD3 ECD, we calculated the distances versus time of the COM of (1) the αβ variable domain, (2) the αβ constant domain, (3) the δε, and (4) γε ECDs, each to a fixed point in the TMR (Cα atom of TCRα:R232 residue) along the vertical axis which is perpendicular to the membrane (S6C Fig). This showed that the TCRαβ variable domain (VαVβ) is the most flexible region of the TCR-CD3 ECD. This flexibility of VαVβ may be required for antigen recognition and initiating allosteric effects onto the rest of the complex [20].

### Atomistic molecular dynamics simulations (ATMD)

The CGMD simulations involved applying an elastic network [21] within each dimer (αβ, δε, γε, ζζ) to maintain their dimeric tertiary/quaternary structures during simulations, hence it restricted conformational changes within the dimers. Therefore, in addition to the ATMD simulations performed for 100 ns using the initial model to confirm the coiling of TCR-CD3 CYRs, we also performed ATMD simulations using the final snapshot from the end of one of the five CGMD simulations by extracting the FL protein along with membrane 1. We converted this snapshot from CG to AT resolution using the backmapping method [22], and further performed three replicates of ATMD simulations for 250 ns each, thereby providing information on the protein-protein interactions and TCR-CD3 dynamics in atomistic (AT) detail. The following criteria were considered for backmapping: (1) the most observed TMR configurational change as shown in Fig 6A, i.e. where the distance between ε(δ) and ζ1 TMRs increased, and (2) penetration of tyrosines into the bilayer, and in particular the penetration of Tyr177 of ε(γ) subunit that was observed most frequently. From these simulations, using the initial model (Fig 1A) as the reference, we calculated the backbone RMSD of the ECD, TMR, and CYRs, and compared them to the initial 250 ns of the CGMD simulations (S7A Fig). We observed that the RMSD of the TMR backbone from the ATMD simulations were similar to that of the CGMD simulations suggesting that its conformation was retained during the ATMD simulations. The backbone RMSD of the CYR in the ATMD simulation was slightly lower compared to that observed in CGMD suggesting that the packing of CYR was refined in the ATMD. Calculation of the RMSD of the ECD backbone in ATMD showed distinct values at early stages of the simulation, confirming the potential of the TCR-CD3 ECD to adopt different conformations (as shown in S5A Fig).

During the ATMD simulations, the protein-lipid interactions observed in CGMD simulations were retained. The loosening between ζ1 and CD3ε(δ) TMRs, seen in CGMD simulations was also maintained throughout the ATMD simulations suggesting that this conformation may be an energetically favourable configuration for the TCR-CD3. This loosening between ζ1 and CD3ε(δ) TMRs brought CD3δ TMR closer to TCRβ TMR as observed in CGMD simulations (Fig 6A and S5C Fig). Although ζ1 and CD3ε(δ) TMRs made minimal contact, they interacted via their extracellular segments and CYRs. ζ1 made no contact with the CD3γε dimer, while ζ2 interacted only with CD3γ via its TMR and CYR (S7B Fig). Consistent with the CGMD simulations, ATMD simulations showed the interactions of the DE loop in the TCRα constant domain and of the FG loop in the TCRβ constant domain with CD3δε and CD3γε ECDs respectively. They also revealed new interactions of Vα and Vβ with the ECDs of CD3δε and CD3γε respectively (Fig 6B and S7C Fig). In addition to the contacts revealed by the cryo-EM study [4], few more residues of TCRβ constant domain (βQ137, βK138) were found to interact with the CD3δ ECD (Fig 6B and S7C Fig). Furthermore, our ATMD simulations showed that the α-helical regions in the CYRs of ζζ were retained. Additionally, despite starting as unstructured regions, the CD3δ, ε, γ CYRs tended to form short α-helices in all ATMD simulations (S8 Fig).

ATMD of the TCR-CD3 complex in the membrane also revealed that, in the outer membrane leaflet, POPC and POPE headgroups were pulled toward the hydrophobic region of the membrane to ionically interact with anionic residues of the CD3 and ζ TMRs (Fig 6C). From all ATMD simulations combined, we calculated the number of contacts between the nitrogen atoms of all outer leaflet lipid headgroups i.e., POPC, POPE, DPSM, and the anionic residues of the CD3 and ζ TMRs. We found that the nitrogen atoms of DPSM made negligible number of contacts, POPC made some contacts, while POPE dominated interaction. The CD3γε TMR induced the most pulling of POPC/POPE headgroups into the membrane followed by the CD3δε and ζζ TMRs (Fig 6D and 6E). Moreover, the pulling down of lipid headgroups resulted in extracellular water solvating some charged residues of the TCR-CD3 TMR, thereby leading to a local membrane deformation in the outer membrane leaflet. Previous MD studies proposed that this occurs prior to TCR-CD3 assembly [23] whereas our studies suggest that this occurrence is also a feature of the entire TCR-CD3 complex.

## Discussion

The CYRs of the CD3 and ζ subunits are essential to mediate T cell activation, as pMHC binding induces tyrosine phosphorylation of their ITAMs by LCK and intracellular signal propagation by the ZAP-70 tyrosine kinase [7]. Biochemical and biophysical studies have suggested that the CYRs of the CD3ε and ζ subunits of a non-stimulated TCR-CD3 complex interact with the inner leaflet of the plasma membrane [9,10]. These data may imply that TCR-CD3 ligation induces dissociation of the CYRs from the inner leaflet of the membrane [11,24]. This dissociation allows augmented ITAM access by active LCK [13] followed by sustained ITAM phosphorylation leading to activation of ZAP-70 to propagate intracellular signalling [7]. Recent data suggest that allosterically-induced conformational changes triggered by pMHC binding to TCR-CD3 facilitate ITAM exposure [20]. It has also been proposed that the dissociation of CYRs could occur by ITAM phosphorylation due to an increase in their net electronegative charge. However, the clusters of positively charged amino acids strongly interacting with the membrane might oppose this dissociation mechanism [8]. This dissociation could perhaps be facilitated by protein kinase binding to phosphorylated ITAMs.

Our study shows that the CYRs of CD3δε, CD3γε and ζζ dimers exhibit a coiled and interlaced conformation forming contacts with each other. This coiled conformation of the tails allowed some cytoplasmic tyrosines to penetrate into the membrane whilst the sidechains of some other cytoplasmic tyrosines were hidden within the CYR coiled conformation. The transient exposure of these hidden ITAM tyrosines to intracellular solvent during our simulations supports findings that resting T cells can undergo basal phosphorylation [25,26]. Moreover, our simulations suggest that the network of protein-protein interactions among the CYRs reduce the solvent accessibility surface area, thereby reducing the probability of ITAM phosphorylation in its resting state. Therefore, an allostery-based stimulation of conformational changes in the TCR-CD3 which alters the interactions of ζζ TMR with the rest of the TCR-CD3 as seen in our simulations can potentially contribute to the unbinding of ζζ CYRs from CD3 CYRs and further increase the exposure of their ITAMs for phosphorylation. This mechanism is consistent with our recent data suggesting that TCR engagement with pMHC leads to ζζ loosening relative to the rest of the complex [20]. Our simulations suggest that the TCR-CD3 CYRs undergo coiling, thereby not allowing all ITAM tyrosines to penetrate the membrane. As a result, the ζ subunits containing the lengthiest CYRs with the highest number of ITAM tyrosines are more likely to expose their ITAMs, compared to the CD3 CYRs. In addition, the total number of tyrosines in the ζζ CYRs is higher compared to the number of tyrosines present in all CD3 CYRs combined, potentially explaining why ζζ plays a major role in signal transduction. This is in agreement with a previous study that proposed that ζζ ITAM multiplicity can enhance signalling [27].

Recent cryo-EM studies revealed most of the TCR-CD3 quaternary structure in a detergent environment, i.e. its TMRs and ECDs, but not its CYRs [4]. This allowed us to employ molecular modelling to complete the TCR-CD3 structure and simulate its dynamics in a bilayer that closely mimics the lipid composition of a TCR-CD3 activation domain in a T cell plasma membrane [14]. We observed in our simulations that the TCR-CD3 conformation was somewhat divergent from the cryo-EM structure, indicating that its conformation may differ in a membrane environment compared to that obtained in a detergent environment. Our simulations suggested an alteration in the TMR configuration where the ζζ and CD3δε dimers lost contact thereby bringing CD3δ and TCRβ TMRs closer than observed in the cryo-EM structure. Moreover, we identified possible conformations of the TCR-CD3 ECD and interactions of VαVβ with CD3 ECDs indicating flexibility of the antigen-binding domain of the TCRαβ, in line with studies that identified allosteric sites in the TCRαβ constant domains [2,20,28–31].

This work also demonstrates that the TCR-CD3 creates a unique lipid fingerprint in the membrane by forming selective interactions with anionic headgroups of PIP lipids. This is in agreement with experimental findings that have suggested that PIP lipids regulate membrane dynamics and TCR-CD3 activation [32]. Such unique membrane footprints have also been suggested for other membrane proteins [33]. Our simulations show that PIP lipids interact strongly with the BRS of CD3ε and ζ subunits in the intracellular region, consistent with findings suggesting that BRS mediate interaction with PIPs and modulate signalling [9,10]. Moreover, the BRS of CD3ε is suggested to serve as a docking site for LCK [34,35] whereas that of ζ is suggested to help localise TCRs at the immunological synapse [10]. Similar cationic patches were observed in the juxtamembrane regions of receptor tyrosine kinases [36–38] and other signalling receptors such as the integrin-talin complexes [39,40] where they were shown to play critical functions in receptor activation by interacting with anionic lipids.

In this study, we also show that the cationic residues that are situated at the interface of the TMR and CYR of the TCR-CD3 can maintain an anionic lipid environment around TCR-CD3 in the absence of the CYR. This suggests that the TCR-CD3 complex can maintain an anionic environment in its vicinity, albeit smaller, even when its cytoplasmic tails are not associated with the membrane. The positively charged regions of the cytoplasmic tails enhance the formation of a distinct annulus of PIP lipids around the TCR-CD3. The triggering of PIP clustering may further create a suitable lipid environment for recruiting peripheral proteins such as LCK. Mutation studies also showed that the LCK-SH2 domain interacts with PIP lipids via a cationic patch at K182 and R184, which is distinct from its phospho-tyrosine binding site [41]. The SH2 domain of ZAP-70 was also found to bind to PIP_2_ headgroups and phospho-tyrosines independently. It was also found that these SH2 domains cannot associate with stimulated TCRs when their lipid-binding site is mutated [42] suggesting that lipid interaction of SH2 domains could be critical to their association with the TCR-CD3. Therefore, the formation of an anionic lipid environment enriched in PIPs around the TCR-CD3 shown in our study may be key for its interactions with LCK and other SH2 domain-containing protein kinases.

In summary, we propose the first molecular model of the entire resting TCR-CD3 complex inserted in an asymmetric bilayer containing lipid types found in the TCR-CD3 activation domain. Our dynamic model (S2 Movie) suggests conformational changes in the TCR-CD3, membrane penetration of some ITAM tyrosines and selective interactions of the TCR-CD3 with cholesterol and anionic headgroups, especially those of PIP lipids whose interactions were enhanced in the presence of CD3 and ζ CYRs. The clustering of PIP lipids around TCR-CD3 may facilitate the initial interaction of its CYRs with LCK, participate in TCR-CD3 clustering and aid in the spatial organization of the immunological synapse. Therefore, our findings can lead to further studies, e.g. involving mutations in the CYRs to alter protein-lipid interactions, to better understand the molecular mechanism of TCR-CD3 activation and signalling.

### Limitations

It is important to consider possible limitations of the simulations performed in this study. Given the lack of structural information on the arrangement of the TCR-CD3 CYR, we modelled this region in an extended conformation perpendicular to the membrane. This allowed us to avoid any bias in inter-chain contacts at the beginning of our simulations. For our CGMD simulations, we used the Martini forcefield. As a result, the structured regions of the ECD and TMR were restrained to maintain their tertiary structure as observed in the cryo-EM structure (PDB:6JXR). Note that these restraints were applied only within each dimer and not between dimers to allow inter-dimeric relaxation as the TCR-CD3 is a complex of four dimers. This limitation was partly addressed by the fact that we employed a multi-scale approach in which we backmapped the final structural configuration of the TCR-CD3 along with the membrane to AT resolution and further simulated this. From these ATMD simulations, we assessed the stability of the different TCR-CD3 domains, which was found to be maintained. It is also suggested that CGMD simulations with the Martini forcefield may exaggerate protein-protein interactions. Therefore, to confirm that the TCR-CD3 CYR forms coiled conformations, we also conducted ATMD simulations starting from the same initial model used at the beginning of our CGMD simulations (with the CYRs in an extended conformation). These ATMD simulations also showed the CYRs coiling, supporting our CGMD simulations. Additionally, the conformations that we observed are in agreement with experimental data suggesting that the TCR-CD3 CYRs interact with the membrane along with certain tyrosines e.g. Tyr177 of CD3ε which were shown to penetrate the membrane [11]. The fact that our CGMD simulations agree with experimental findings provides a further validation for our study on the TCR-CD3 dynamics.

## Methods

### Molecular modelling

The cryo-EM structure (PDB:6JXR) [4] was used as a template to obtain the complete TCR-CD3 model. Sequences of each subunit were obtained from UniProtKB (uniprot.org): ζ:P20963, δ:P04234, ε:P07766, γ:P09693, α:A0A0B4J271, β:P0DSE2. Modeller 9.2 was used to model the entire TCR-CD3 complex [43,44] (S1A Fig) along with UCSF Chimera [45]. Hydrogen atoms were added to the model and topologies were generated using the Charmm36 forcefield [46] and Gromacs 2016 [47].

### Coarse-grained molecular dynamics (CGMD) simulations

The structural models were coarse-grained using the martinize script. CGMD simulations were conducted using Gromacs version 5.0 with the Martini 2.2 forcefield [48,49]. The TCR-CD3 is comprised of the αβ, δε, γε, ζζ dimers non-covalently bonded with each other. To replicate this and to avoid restraints between each dimer, an elastic network model [21] with a lower cut-off distance of 0 nm, an upper cut-off distance of 0.7 nm and force constant of 1000 kJ/mol/nm^2^ was applied only between subunits within each dimer to maintain their dimeric tertiary/quaternary structures. This was done by martinizing each dimer separately and then concatenating their coordinates. Note also that there was no elastic network restraints between the unstructured regions of the tails, but only within the small helical regions in the ζζ tails. The tails of each subunit were modelled sufficiently far away from each other to observe intracellular dynamics without any restraints. The protein complex was then placed in a simulation box and inserted into a complex asymmetric bilayer using the *Insane* tool [50]. The concentration of lipid headgroups in both membrane 1 and 2 was the same (Table 1) and is based on lipidomics studies of TCR-CD3 activation domains [14]. Membrane 1 lipids each contained mono-unsaturated and saturated acyl chains whereas membrane 2 contained a wider variety and a higher percentage of poly-unsaturated lipid acyl chains (S2A Fig) which was also derived from the same lipidomics study. CG waters were added, and the system was neutralized with 0.15M of Na+ and Cl- ions. They were energy minimized using the steepest descent algorithm followed by equilibration with the protein backbone particles position-restrained for 2.5 ns. The equilibrated system was used to generate systems with differing initial velocities for five production simulations run for 5 μs each with 20 fs time-step. Equilibration and production simulations were conducted using the NPT ensemble. Every frame in each production simulation was generated at 200 ps intervals. The semi-isotropic barostat and thermostat used for CGMD production simulations were Parinello-Rahman (1 bar) [51] and V-rescale (323 K) [52], respectively. A compressibility of 3x10^-4^ bar^-1^ was used.

### Atomistic molecular dynamics (ATMD) simulations

ATMD simulations (100 ns x 3 replicates) were initially performed starting from the same initial model as CGMD simulations to confirm the coiled conformation of the TCR-CD3 CYR. This was set up using Charmm-GUI [53] using the membrane 1 composition, TIP3P water model and neutralized with 0.15M Na+ and Cl- ions. Energy minimization using the steepest descent algorithm was conducted until forces converged to 1000 kJ/mol/nm^2^ followed by a 6-step semi-isotropic equilibration at 323 K where position restraints in the system were gradually released. Production simulations were run for 100 ns x 3 replicates using the Nose-Hoover thermostat (323 K) and Parrinello-Rahman semi-isotropic barostat (1 bar) with a compressibility of 4.5x10^-5^ bar^-1^ and frames were generated every 100 ps. The LINCS algorithm was used to constrain hydrogen bond lengths and the Particle Mesh Ewald algorithm defined long-range electrostatics.

A frequently observed conformation in which (1) ζ1 and CD3ε(δ) TMRs had undergone loosening and (2) ITAM Tyr177 of CD3ε(γ) was inserted in the membrane, was extracted from the end of a CGMD simulation and backmapped [22] to AT resolution. Gromacs 2016 with the Charmm36 forcefield [46] was used for backmapping. Lipid parameters were obtained from Charmm-GUI [53,54]. Before performing ATMD, the simulation box size was reduced along the vertical (Z) axis to prevent simulating excess solvent particles, thus minimizing the computational cost. The protein-lipid conformation was retained during the resizing of Z axis of the simulation box. The TIP3P water model was used along with 0.15M of Na+ and Cl- ions to neutralize the system. The system was energy minimized with the steepest descent algorithm followed by a step-wise (0.25→1→1.5→1.8→1.9→2 fs time-step) NPT equilibration with the protein backbone position-restrained. The final equilibration step was conducted for 2 ns with a 2 fs time-step. The equilibrated system was then used to generate systems with different initial velocities for three repeat production simulations each of which were run for 250 ns with a 2 fs time-step. Every frame in each simulation was generated at 40 ps intervals. The V-rescale thermostat (323 K) and Parrinello-Rahman semi-isotropic barostat (1 bar) [51] was used with a compressibility of 4.5x10^-5^ bar^-1^. The LINCS algorithm [55] applied constraints on all bond lengths and the Particle Mesh Ewald algorithm [56] defined long-range electrostatics. A summary of simulations is shown in Table 2. The number of each lipid type used in our simulations is shown in Table 3.

**Table 2:** Summary of simulations conducted in this study.

Simulations ^a^	Resolution	Particles	Simulation box (X × Y × Z axis)	Duration	Replicas
TMO (memb 1)^b^	CG	26728	17 × 17 × 10 nm	5 μs	5
ECTM (memb 1)^c^	CG	54106	17 × 17 × 21 nm	5 μs	5
FL (memb 1)^d^	CG	196303	25 × 25 × 38 nm	5 μs	5
FL (memb 1)^d^	AT	1517173	20 × 20 × 37 nm	100 ns	3
FL (memb 1)^d^	AT (backmapped)	1145375	25 × 25 × 21 nm	250 ns	3
FL (memb 2)^d^	CG	196030	25 × 25 × 38 nm	5 μs	5

^a^ All simulations were conducted in an asymmetric complex bilayer with the same lipid headgroup composition (see Table 1)

^b^ TMO: Simulations with transmembrane domain only.

^c^ ECTM: Simulations of the extracellular and transmembrane domains only.

^d^ FL: Simulations with full-length subunits i.e. complete TCR-CD3.

(Memb 1/Memb 2): See S2A Fig for difference in lipid acyl chain composition

**Table 3:** Number of lipids in each leaflet of the membranes and the number of CG water particles used in each simulation in this study.

	TMO/ECTM (XY plane = 17 nm^2^)		FL (XY plane = 25 nm^2^) (membrane 1)		FL (XY plane = 25 nm^2^) (membrane 2)	
	Outer leaflet	Inner leaflet	Outer leaflet	Inner leaflet	Outer leaflet	Inner leaflet
POPC	254	50	502	99	351	69
PIPC	-	-	-	-	150	29
POPE	50	201	100	399	20	99
PAPE	-	-	-	-	20	99
DOPE	-	-	-	-	60	199
DPSM	101	-	201	-	180	-
PNSM	-	-	-	-	20	-
Chol	101	100	201	199	200	199
POPS	-	100	-	199	-	119
DOPS	-	-	-	-	-	79
PIP2	-	40	-	79	-	79
PIP3	-	10	-	19	-	19
Water particles	TMO = 13799 ECTM = 38666		165366		166370	

### Analysis

Calculation of all protein-lipid and protein-protein contacts in the CGMD simulations used a 0.55 nm distance cut-off to define a contact. The same cut-off value was used to calculate contacts of protein residues with hydrophobic lipid acyl chains. Similarly, all contact analyses for ATMD simulations used a 0.4 nm cut-off. All interaction profiles represent merged data from all simulation repeats and were performed using *gmx mindist* command and in-house python scripts. Residence times of lipids were calculated using the PyLipid tool (https://github.com/wlsong/PyLipID). To calculate clusters of TCR-CD3 cytoplasmic conformations, and lipid densities around the protein, the trajectories of all simulation repeats were concatenated using *gmx trjcat* and the protein orientation was fixed in the center using *gmx trjconv*. The *gmx densmap* and *gmx xpm2ps* commands were used to produce lipid density images. The clustering analysis of the cytoplasmic conformations was performed using the *gmx cluster* command using the single linkage method. Clustering analysis was done using a 0.35 nm cut-off for the protein backbone RMSD for every 10^th^ frame i.e. 400 ps. RMSDs and RMSFs were calculated with *gmx rms* and *gmx rmsf* respectively. Distance and radius of gyration analyses were performed using the *gmx distance* and *gmx gyrate* commands respectively. Radial distribution functions were calculated using *gmx rdf*. VMD was used for visualization and rendering. The RMSD trajectory tool of VMD [57] was also used to perform alignments of TMR helices. The electrostatic potential (kT/e) of the entire TCR-CD3 was obtained using the APBS electrostatics tool [18] integrated with VMD. The MUSCLE tool was used to perform multiple sequence alignments [58]. Calculation of the relative height of lipid nitrogen atoms was based on their positions along the vertical (Z) axis i.e. perpendicular to the membrane. For this calculation, the TCR-CD3 orientation was fixed in the center in each ATMD simulation before concatenating all of them. The python script to perform this calculation was obtained from https://github.com/jiehanchong/membrane-depth-analysis. The *gmx do_dssp* command was used to calculate the secondary structure formations in the TCR-CD3 CYRs considering 1 ns intervals from all ATMD simulations combined. Xmgrace (https://plasma-gate.weizmann.ac.il/Grace/) and Matplotlib 3.3 (doi.org/10.5281/zenodo.3948793) were used for plotting.

## Supporting information

S1 Fig Sequences of the full-length subunits of the TCR-CD3 and the conformations of its cytoplasmic region. **(A)** Sequences of full-length subunits of the TCR-CD3 used for modelling. See Methods for Uniprot sequence IDs. Their secondary structure was predicted by the PSIPRED 4.0 server. The extracellular and intracellular residues modelled in this study are shown in boxes and underlined respectively. **(B)** Snapshot from one of the CGMD and ATMD simulations at simulation time (t) = 100 ns, starting from the same initial model (shown on the left). **(C)** TCR-CD3 cytoplasmic conformations grouped into clusters using a 3.5 Å RMSD cut-off, related to Fig 2A. The clusters containing the highest number of structures indicate the most stable conformation of the TCR-CD3 cytoplasmic region in our simulations. In (B) and (C), the structure is coloured by subunit: ζ:red, δ:blue, ε:yellow, γ:green, α:silver, β:black.
(PDF)

S2 Fig Membrane composition and interaction of TCR-CD3 with lipids. **(A)** Unsaturation levels of each leaflet of membranes 1 and 2 (top) and their lipid compositions (below), see also Tables 1 and 3. **(B)** Sequences of the ECTM (ectodomain and transmembrane), and TMO (transmembrane only) systems used for the CGMD simulations. The TMO sequences are a subset of the ECTM sequences, related to Fig 5A. **(C)** Extracellular view of the density of each lipid type in the membrane when the orientation of the TCR-CD3 is fixed in the center, related to Fig 5B. The colour gradient scale for each density displays the number of lipids corresponding to the minimum and maximum value. **(D)** Radial distribution functions of all lipid types in membrane 1 and 2. The different lines for each lipid type represent the RDF for the five repeat simulations that we performed for each system. **(E)** The average number of cholesterol, DPSM, POPC, POPE, and POPS lipids interacting with the TCR-CD3 over 5 μs time from all CGMD simulations of TMO, ECTM, FL systems conducted in membrane 1, related to Fig 5C.
(PDF)

S3 Fig Cholesterol interactions with TCR-CD3, residence times and multiple sequence alignment. **(A)** Comparison of cholesterol interactions with the complete TCR-CD3 (FL), ECTM and TMO simulations. Normalization was done by dividing the contacts of each residue in the TCR-CD3 by the highest number of contacts. Therefore, the value 1 represents the highest contact while 0 represents no contact. The three TCR-CD3 systems: TMO, ECTM, FL, are normalized separately. **(B)** Residence times of cholesterol and anionic lipids shown for membrane 1 and 2. **(C)** The cationic anchor of the TCR-CD3, related to Fig 5E, located at the interface of the TMR and CYR is indicated within a box. **(D)** Multiple sequence alignment of the TMR and juxtamembrane residues of all TCR-CD3 subunits, related to Fig 5E, indicates the conservation of cationic residues at the TMR-CYR interface across different species i.e. Homo sapiens (humans), Rattus norvegicus (rat), Sus Scrofa (pig), Bos taurus (cow), Ovis aries (sheep), and Macaca mulatta (monkey).
(PDF)

S4 Fig Interactions of TCR-CD3 with PIP_2_, PIP_3_, and PS lipids. Interactions of **(A)** PIP_2_, **(B)** PIP_3_, and **(C)** PS with the complete TCR-CD3 (FL), ECTM and TMO simulations. Normalization is done by dividing the contacts of each residue in the TCR-CD3 with a lipid type by the highest number of contacts with that lipid type. Therefore, the value 1 represents the highest contact while 0 represents no contact. The three TCR-CD3 systems: TMO, ECTM, FL, are normalized separately.
(PDF)

S5 Fig Conformational changes within the TCR-CD3. **(A)** Backbone RMSD of the TCR-CD3 ECD, TMR, and CYR in membrane 1 and 2. **(B)** Backbone RMSF of the complete TCR-CD3 in the CGMD and ATMD simulations. A structure extracted at 1 μs was used a reference for this RMSF calculation. **(C)** Extracellular view of five TMR snapshots aligned from the end of the TMO, ECTM, FL simulations indicating the frequency of ζ1-ε(δ) dissociation compared to the cryo-EM TMR structure (PDB:6JXR). **(D)** Distance between the center of mass of ζ1 and of ε(δ) subunits, and **(E)** distance between the center of mass of δ and of β subunits in all simulations systems over 5 μs compared to their initial distances calculated from the cryo-EM structure.
(PDF)

S6 Fig Inter-chain interactions of the complete TCR-CD3 in the coarse-grained simulations. Normalized full-length inter-chain interactions of **(A)** the ζζ dimer with the CD3δε and CD3γε dimers, and **(B)** the TCRαβ dimer with the ζζ, CD3δε, and CD3γε dimers in CGMD simulations. Normalization is done by dividing the contacts of each residue by the highest number of contacts within each dimer. **(C)** Distance of the center of mass of the TCRαβ variable domain (VαVβ), the TCRαβ constant domain (CαCβ), the CD3δε, and CD3γε ectodomains (EC), each to the Cα atom of TCRα:R232 residue in the TMR along the vertical (Z) axis. The smoothened lines are a polynomial regression to the 10^th^ degree of the distances versus time.
(PDF)

S7 Fig RMSD of the TCR-CD3 and its inter-chain interactions in the atomistic simulations. **(A)** Backbone RMSD of the ECD, TMR, and CYR of the TCR-CD3 in the initial 250 ns of the CGMD and the backmapped ATMD simulations. Backbone RMSDs during the last 500 ns of CGMD are also shown for comparison. The initial model of the TCR-CD3, as seen in Fig 1, was used as a reference for this calculation. **(B)** Normalized full-length inter chain interactions of the ζζ dimer with the CD3δε and CD3γε dimers, and **(C)** the TCRαβ dimer with the ζζ, CD3δε, and CD3γε dimers in ATMD simulations. Normalization is done by dividing the contacts of each residue by the highest number of contacts within each dimer.
(PDF)

S8 Fig α-helix formation in the TCR-CD3 CYR in the backmapped atomistic simulations. Number of α-helix forming residues in the CD3 and ζ CYRs versus time from all three backmapped ATMD simulations that were run for 250 ns (left). Location of cytoplasmic α-helices relative to the ITAM tyrosines of the respective subunits (right). The ITAM tyrosines and α-helix forming residues in the CD3 subunits observed at the end of 250 ns in simulation-3 are labelled. The entire protein is shown using the surface representation, the ITAM tyrosines are represented as black ball and sticks, and the subunits are shown in cartoon representation and coloured as follows: ζ:red, δ:blue, ε:yellow, γ:green (right).
(PDF)

S1 Movie CGMD simulation showing the coiling of cytoplasmic tails followed by membrane penetration of Y177 (yellow spheres) sidechain of the CD3ε(γ) subunit. Cationic residues in the cytoplasmic region are shown as transparent blue spheres while ITAM tyrosines are shown as solid coloured spheres. The TCR-CD3 backbone atoms are connected by dynamic bonds shown in white. Membrane lipids are represented by coloured lines (POPC:brown, POPE:purple, POPS:magenta, PIP_2_:orange, PIP_3_:red, cholesterol:cyan, DPSM:green), except phosphate groups which are shown as small opaque spheres. The total simulation time shown in the video is 73 ns starting from t = 0 where the cytoplasmic tails are modelled in an extended conformation.
(MP4)

S2 Movie An overall view of the TCR-CD3 dynamics and interactions with cholesterol and PIP lipids in a coarse-grained simulation. The backbone of the entire TCR-CD3 is shown as black dynamic bonds and a grey surface. The phosphate groups of membrane lipids are shown as small spheres coloured in tan. Cholesterol, PIP_2_ and PIP_3_ lipids in the vicinity of the TCR-CD3 backbone are shown as cyan spheres, orange surface, and red surface, respectively. ITAM tyrosines are shown as green spheres. The total simulation time observed in the video is 4 μs, starting from 1 μs simulation time.
(MP4)

## References

[1] Courtney AH, Lo WL, Weiss A. TCR Signaling: Mechanisms of Initiation and Propagation. Trends in Biochemical Sciences. Elsevier Ltd; 2018. pp. 108–123. doi: 10.1016/j.tibs.2017.11.008 29269020 PMC5801066

[2] Mariuzza RA, Agnihotri P, Orban J. The structural basis of T-cell receptor (TCR) activation: An enduring enigma. Journal of Biological Chemistry. American Society for Biochemistry and Molecular Biology Inc.; 2019. pp. 914–925. doi: 10.1074/jbc.REV119.009411 31848223 PMC6983839

[3] Call ME, Pyrdol J, Wiedmann M, Wucherpfennig KW. The organizing principle in the formation of the T cell receptor-CD3 complex. Cell. 2002;111: 967–979. doi: 10.1016/s0092-8674(02)01194-7 12507424 PMC3420808

[4] Dong D, Zheng L, Lin J, Zhang B, Zhu Y, Li N, et al. Structural basis of assembly of the human T cell receptor–CD3 complex. Nature. 2019;573: 546–552. doi: 10.1038/s41586-019-1537-0 31461748

[5] Kuhns MS, Davis MM. Disruption of Extracellular Interactions Impairs T Cell Receptor-CD3 Complex Stability and Signaling. Immunity. 2007;26: 357–369. doi: 10.1016/j.immuni.2007.01.015 17368054

[6] Garcia KC, Degano M, Speir JA, Wilson IA. Emerging principles for T cell receptor recognition of antigen in cellular immunity. Rev Immunogenet. 1999;1: 75–90. 11256574

[7] Acuto O, Bartolo V Di, Michel F. Tailoring T-cell receptor signals by proximal negative feedback mechanisms. Nature Reviews Immunology. Nature Publishing Group; 2008. pp. 699–712. doi: 10.1038/nri2397 18728635

[8] Sigalov AB, Aivazian DA, Uversky VN, Stern LJ. Lipid-binding activity of intrinsically unstructured cytoplasmic domains of multichain immune recognition receptor signaling subunits. Biochemistry. American Chemical Society; 2006;45: 15731–15739. doi: 10.1021/bi061108f 17176095 PMC2528957

[9] DeFord-Watts LM, Tassin TC, Becker AM, Medeiros JJ, Albanesi JP, Love PE, et al. The Cytoplasmic Tail of the T Cell Receptor CD3 Subunit Contains a Phospholipid-Binding Motif that Regulates T Cell Functions. J Immunol. The American Association of Immunologists; 2009;183: 1055–1064. doi: 10.4049/jimmunol.0900404 19542373 PMC2954055

[10] DeFord-Watts LM, Dougall DS, Belkaya S, Johnson BA, Eitson JL, Roybal KT, et al. The CD3 zeta subunit contains a phosphoinositide-binding motif that is required for the stable accumulation of TCR-CD3 complex at the immunological synapse. J Immunol. NIH Public Access; 2011;186: 6839–47. doi: 10.4049/jimmunol.1002721 21543646 PMC3110614

[11] Xu C, Gagnon E, Call ME, Schnell JR, Schwieters CD, Carman C V, et al. Regulation of T cell receptor activation by dynamic membrane binding of the CD3epsilon cytoplasmic tyrosine-based motif. Cell. 2008;135: 702–13. doi: 10.1016/j.cell.2008.09.044 19013279 PMC2597348

[12] Zimmermann K, Eells R, Heinrich F, Rintoul S, Josey B, Shekhar P, et al. The cytosolic domain of T-cell receptor associates with membranes in a dynamic equilibrium and deeply penetrates the bilayer. J Biol Chem. American Society for Biochemistry and Molecular Biology Inc.; 2017;292: 17746–17759. doi: 10.1074/jbc.M117.794370 28893902 PMC5663876

[13] Nika K, Soldani C, Salek M, Paster W, Gray A, Etzensperger R, et al. Constitutively active lck kinase in T cells drives antigen receptor signal transduction. Immunity. Cell Press; 2010;32: 766–777. doi: 10.1016/j.immuni.2010.05.011 20541955 PMC2996607

[14] Zech T, Ejsing CS, Gaus K, De Wet B, Shevchenko A, Simons K, et al. Accumulation of raft lipids in T-cell plasma membrane domains engaged in TCR signalling. EMBO J. 2009;28: 466–476. doi: 10.1038/emboj.2009.6 19177148 PMC2657588

[15] Buchan DWA, Jones DT. The PSIPRED Protein Analysis Workbench: 20 years on. Nucleic Acids Res. 2019; doi: 10.1093/nar/gkz297 31251384 PMC6602445

[16] Duchardt E, Sigalov AB, Aivazian D, Stern LJ, Schwalbe H. Structure induction of the T-cell receptor ζ-chain upon lipid binding investigated by NMR spectroscopy. ChemBioChem. WILEY-VCH Verlag; 2007;8: 820–827. doi: 10.1002/cbic.200600413 17410622

[17] Shen MY, Sali A. Statistical potential for assessment and prediction of protein structures. Prot Sci. Wiley-Blackwell; 2006;15: 2507–24. doi: 10.1110/ps.062416606 17075131 PMC2242414

[18] Baker NA, Sept D, Joseph S, Holst MJ, McCammon JA. Electrostatics of nanosystems: Application to microtubules and the ribosome. Proc Natl Acad Sci. National Academy of Sciences; 2001;98: 10037–10041. doi: 10.1073/pnas.181342398 11517324 PMC56910

[19] Li L, Shi X, Guo X, Li H, Xu C. Ionic protein–lipid interaction at the plasma membrane: what can the charge do? Trends Biochem Sci. Elsevier Current Trends; 2014;39: 130–140. doi: 10.1016/J.TIBS.2014.01.002 24534649

[20] Lanz A-L, Masi G, Porciello N, Cohnen A, Cipria D, Prakaash D, et al. Allosteric activation of T-cell antigen receptor signalling by quaternary structure relaxation. Cell Rep. 2021; 36. doi: 10.1016/j.celrep.2021.109375 34260912 PMC8293630

[21] Periole X, Cavalli M, Marrink S-JJ, Ceruso MA. Combining an elastic network with a coarse-grained molecular force field: Structure, dynamics, and intermolecular recognition. J Chem Theory Comput. American Chemical Society; 2009;5: 2531–2543. doi: 10.1021/ct9002114 26616630

[22] Wassenaar TA, Pluhackova K, Böckmann RA, Marrink SJ, Tieleman DP. Going backward: A flexible geometric approach to reverse transformation from coarse grained to atomistic models. J Chem Theory Comput. American Chemical Society; 2014;10: 676–690. doi: 10.1021/ct400617g 26580045

[23] Sharma S, Juffer AH. An atomistic model for assembly of transmembrane domain of T cell receptor complex. J Am Chem Soc. 2197th ed. American Chemical Society; 2013;135: 2188–2197. doi: 10.1021/ja308413e 23320396

[24] Gil D, Schamel WWA, Montoya M, Sá Nchez-Madrid F, Alarcó B, Sánchez-Madrid F, et al. Recruitment of Nck by CD3 Reveals a Ligand-Induced Conformational Change Essential for T Cell Receptor Signaling and Synapse Formation. Cell. Cell Press; 2002;109: 901–912. doi: 10.1016/s0092-8674(02)00799-7 12110186

[25] Schoenborn JR, Tan YX, Zhang C, Shokat KM, Weiss A. Feedback circuits monitor and adjust basal Lck-dependent events in T cell receptor signaling. Sci Signal. American Association for the Advancement of Science; 2011;4: ra59–ra59. doi: 10.1126/scisignal.2001893 21917715 PMC4080844

[26] Sjölin-Goodfellow H, Frushicheva MP, Ji Q, Cheng DA, Kadlecek TA, Cantor AJ, et al. The catalytic activity of the kinase ZAP-70 mediates basal signaling and negative feedback of the T cell receptor pathway. Sci Signal. American Association for the Advancement of Science; 2015;8: ra49. doi: 10.1126/scisignal.2005596 25990959 PMC4445242

[27] Love PE, Hayes SM. ITAM-mediated Signaling by the T-Cell Antigen Receptor. Cold Spring Harb Perspect Biol. 2010;2. doi: 10.1101/cshperspect.a002485 20516133 PMC2869518

[28] Natarajan K, McShan AC, Jiang J, Kumirov VK, Wang R, Zhao H, et al. An allosteric site in the T-cell receptor Cβ domain plays a critical signalling role. Nat Commun. Nature Publishing Group; 2017;8. doi: 10.1038/ncomms15260 28508865 PMC5440810

[29] Rangarajan S, He Y, Chen Y, Kerzic MC, Ma B, Gowthaman R, et al. Peptide–MHC (pMHC) binding to a human antiviral T cell receptor induces long-range allosteric communication between pMHC- and CD3-binding sites. J Biol Chem. American Society for Biochemistry and Molecular Biology Inc.; 2018;293: 15991–16005. doi: 10.1074/jbc.RA118.003832 30135211 PMC6187629

[30] Martin-Blanco N, Blanco R, Alda-Catalinas C, Bovolenta ER, Oeste CL, Palmer E, et al. A window of opportunity for cooperativity in the T Cell Receptor. Nat Commun. Nature Publishing Group; 2018;9: 2618. doi: 10.1038/s41467-018-05050-6 29976994 PMC6033938

[31] Beddoe T, Chen Z, Clements CS, Ely LK, Bushell SR, Vivian JP, et al. Antigen Ligation Triggers a Conformational Change within the Constant Domain of the αβ T Cell Receptor. Immunity. 2009;30: 777–788. doi: 10.1016/j.immuni.2009.03.018 19464197

[32] Chouaki-Benmansour N, Ruminski K, Sartre AM, Phelipot MC, Salles A, Bergot E, et al. Phosphoinositides regulate the TCR/CD3 complex membrane dynamics and activation. Sci Rep. Nature Publishing Group; 2018;8. doi: 10.1038/s41598-018-23109-8 29563576 PMC5862878

[33] Corradi V, Sejdiu BI, Mesa-Galloso H, Abdizadeh H, Noskov SY, Marrink SJ, et al. Emerging Diversity in Lipid-Protein Interactions. Chem Rev. American Chemical Society; 2019;119: 5775–5848. doi: 10.1021/acs.chemrev.8b00451 30758191 PMC6509647

[34] Li L, Guo X, Shi X, Li C, Wu W, Yan C, et al. Ionic CD3–Lck interaction regulates the initiation of T-cell receptor signaling. Proc Natl Acad Sci U S A. National Academy of Sciences; 2017;114: E5891–E5899. doi: 10.1073/pnas.1701990114 28659468 PMC5530670

[35] Hartl FA, Beck-Garcìa E, Woessner NM, Flachsmann LJ, Cárdenas RMHV, Brandl SM, et al. Noncanonical binding of Lck to CD3ε promotes TCR signaling and CAR function. Nat Immunol. Nature Research; 2020;21: 902–913. doi: 10.1038/s41590-020-0732-3 32690949

[36] Hedger G, Sansom MSP, Koldsø H. The juxtamembrane regions of human receptor tyrosine kinases exhibit conserved interaction sites with anionic lipids. Sci Rep. Macmillan Publishers Limited. All rights reserved; 2015;5: 9198. doi: 10.1038/srep09198 25779975 PMC4361843

[37] Chavent M, Karia D, Kalli AC, Seiradake E, Jones EY, Sansom Correspondence MSP. Interactions of the EphA2 Kinase Domain with PIPs in Membranes: Implications for Receptor Function. Structure. 2018;26: 1025–34. doi: 10.1016/j.str.2018.05.003 29887500 PMC6039763

[38] Michailidis IE, Rusinova R, Georgakopoulos A, Chen Y, Iyengar R, Robakis NK, et al. Phosphatidylinositol-4,5-bisphosphate regulates epidermal growth factor receptor activation. Pflugers Arch Eur J Physiol. Springer-Verlag; 2011;461: 387–397. doi: 10.1007/s00424-010-0904-3 21107857 PMC3281421

[39] Kalli AC, Campbell ID, Sansom MSP. Conformational Changes in Talin on Binding to Anionic Phospholipid Membranes Facilitate Signaling by Integrin Transmembrane Helices. PLoS Comput Biol. Public Library of Science; 2013;9: e1003316. doi: 10.1371/journal.pcbi.1003316 24204243 PMC3814715

[40] Saltel F, Mortier E, Hytönen VP, Jacquier MC, Zimmermann P, Vogel V, et al. New PI(4,5)P2- and membrane proximal integrin-binding motifs in the talin head control β3-integrin clustering. J Cell Biol. The Rockefeller University Press; 2009;187: 715–731. doi: 10.1083/jcb.200908134 19948488 PMC2806581

[41] Sheng R, Jung DJ, Silkov A, Kim H, Singaram I, Wang ZG, et al. Lipids regulate Lck protein activity through their interactions with the Lck Src homology 2 domain. J Biol Chem. 2016;291: 17639–17650. doi: 10.1074/jbc.M116.720284 27334919 PMC5016160

[42] Park MJ, Sheng R, Silkov A, Jung DJ, Wang ZG, Xin Y, et al. SH2 Domains Serve as Lipid-Binding Modules for pTyr-Signaling Proteins. Mol Cell. 2016;62: 7–20. doi: 10.1016/j.molcel.2016.01.027 27052731 PMC4826312

[43] Fiser A, Šali A. Modeller: Generation and refinement of homology-based protein structure models. Methods Enzym. Academic Press; 2003;374: 461–491. doi: 10.1016/S0076-6879(03)74020-8 14696385

[44] Webb B, Sali A. Protein structure modeling with MODELLER. Methods Mol Biol. Humana Press Inc.; 2014;1137. doi: 10.1007/978-1-4939-0366-5_1 24573470

[45] Pettersen EF, Goddard TD, Huang CC, Couch GS, Greenblatt DM, Meng EC, et al. UCSF Chimera—a visualization system for exploratory research and analysis. J Comput Chem. 2004;25: 1605–12. doi: 10.1002/jcc.20084 15264254

[46] Huang J, MacKerell AD. CHARMM36 all-atom additive protein force field: Validation based on comparison to NMR data. J Comput Chem. 2013;34: 2135–2145. doi: 10.1002/jcc.23354 23832629 PMC3800559

[47] Van Der Spoel D, Lindahl E, Hess B, Groenhof G, Mark AE, Berendsen HJC. GROMACS: Fast, flexible, and free. J Comput Chem. 2005;26: 1701–1718. doi: 10.1002/jcc.20291 16211538

[48] De Jong DH, Singh G, Bennett WFDD, Arnarez C, Wassenaar TA, Schäfer L V., et al. Improved parameters for the martini coarse-grained protein force field. J Chem Theory Comput. American Chemical Society; 2013;9: 687–697. doi: 10.1021/ct300646g 26589065

[49] Marrink SJ, Risselada HJ, Yefimov S, Tieleman DP, De Vries AH. The MARTINI force field: Coarse grained model for biomolecular simulations. J Phys Chem B. American Chemical Society; 2007;111: 7812–7824. doi: 10.1021/jp071097f 17569554

[50] Wassenaar TA, Ingólfsson HI, Böckmann RA, Tieleman DP, Marrink SJ. Computational Lipidomics with *insane*: A Versatile Tool for Generating Custom Membranes for Molecular Simulations. J Chem Theory Comput. American Chemical Society; 2015;11: 2144–2155. doi: 10.1021/acs.jctc.5b00209 26574417

[51] Parrinello M, Rahman A. Polymorphic transitions in single crystals: A new molecular dynamics method. J Appl Phys. AIP; 1981;52: 7182–7190. doi: 10.1063/1.328693

[52] Bussi G, Donadio D, Parrinello M. Canonical sampling through velocity rescaling. J Chem Phys. American Institute of Physics; 2007;126: 14101. doi: 10.1063/1.2408420 17212484

[53] Jo S, Kim T, Iyer VG, Im W. CHARMM-GUI: A web-based graphical user interface for CHARMM. J Comput Chem. John Wiley & Sons, Ltd; 2008;29: 1859–1865. doi: 10.1002/jcc.20945 18351591

[54] Klauda JB, Venable RM, Freites JA, O’Connor JW, Tobias DJ, Mondragon-Ramirez C, et al. Update of the CHARMM All-Atom Additive Force Field for Lipids: Validation on Six Lipid Types. J Phys Chem B. American Chemical Society; 2010;114: 7830–7843. doi: 10.1021/jp101759q 20496934 PMC2922408

[55] Hess B, Bekker H, Berendsen HJC, Fraaije JGEM. LINCS: A linear constraint solver for molecular simulations. J Comput Chem. Wiley-Blackwell; 1997;18: 1463–1472. doi: 10.1002/(SICI)1096-987X(199709)18:12&lt;1463::AID-JCC4&gt;3.0.CO;2-H

[56] Essmann U, Perera L, Berkowitz ML, Darden T, Lee H, Pedersen LG. A smooth particle mesh Ewald method. J Chem Phys. 1995;103: 8577–8593. doi: 10.1063/1.470117

[57] Humphrey W, Dalke A, Schulten K. VMD: visual molecular dynamics. J Mol Graph. Elsevier; 1996;14: 33–8, 27–8. doi: 10.1016/0263-7855(96)00018-5 8744570

[58] Edgar RC. MUSCLE: A multiple sequence alignment method with reduced time and space complexity. BMC Bioinformatics. BMC Bioinformatics; 2004;5. doi: 10.1186/1471-2105-5-113 15318951 PMC517706

