# Supplementary figures and images for: Multi-scale simulations of the T cell receptor reveal its lipid interactions, dynamics and the arrangement of its cytoplasmic region
Source: PLoS Comput Biol. 2021 Jul 19;17(7):e1009232. doi: 10.1371/journal.pcbi.1009232 (PMC8321403; doi:10.1371/journal.pcbi.1009232)

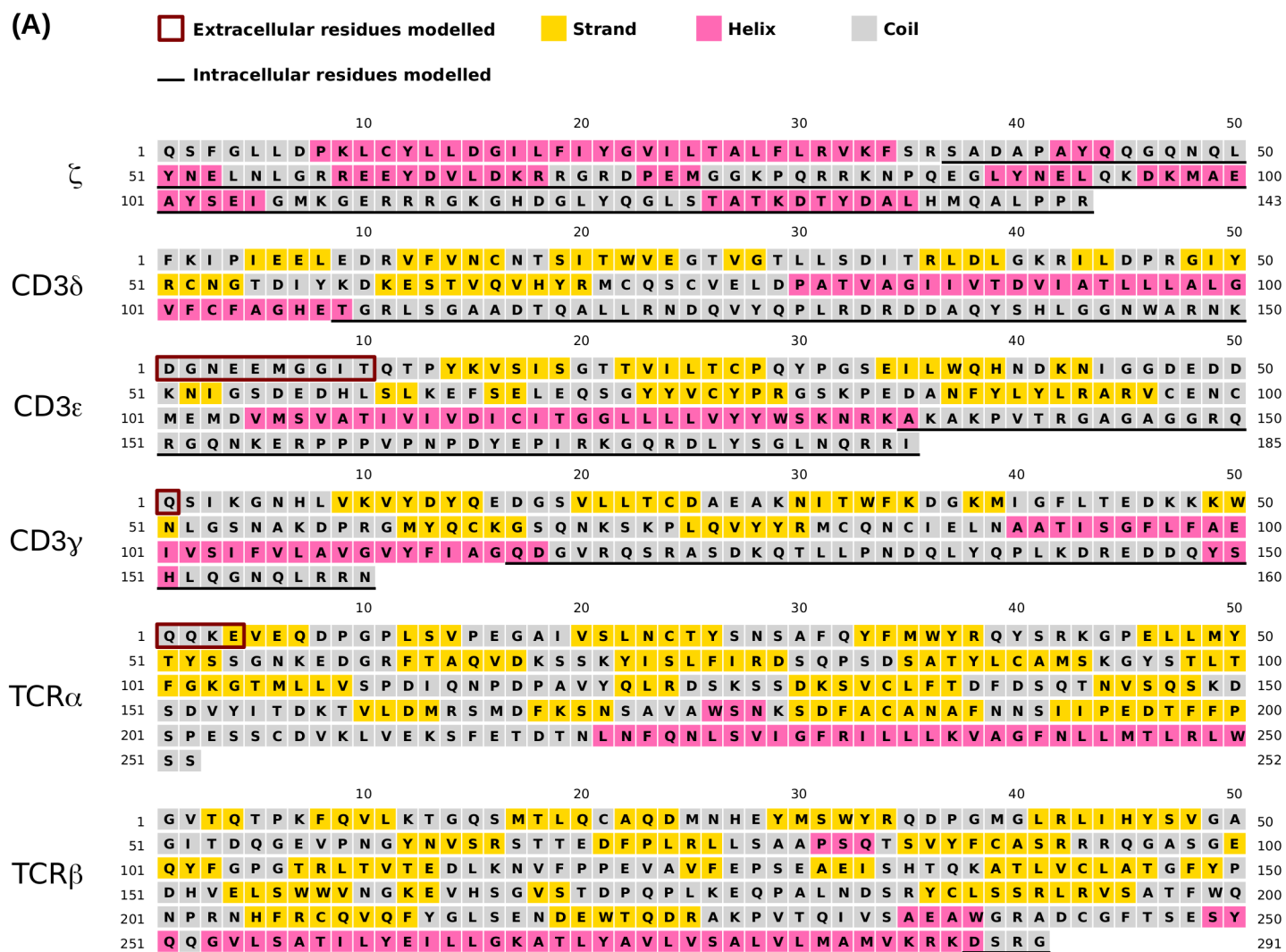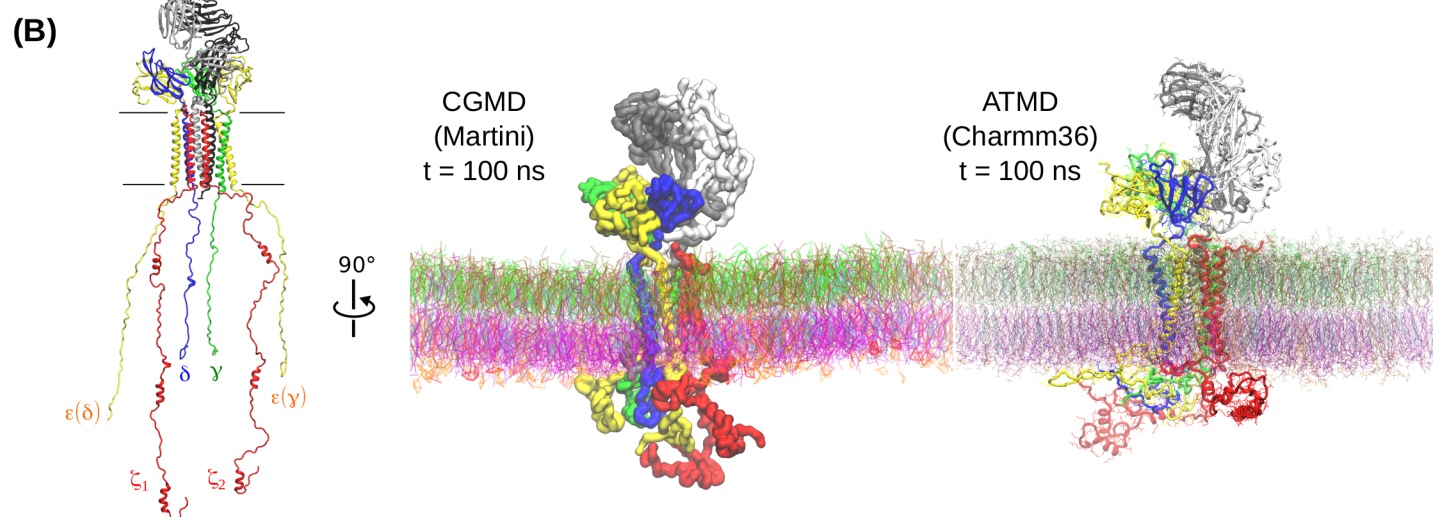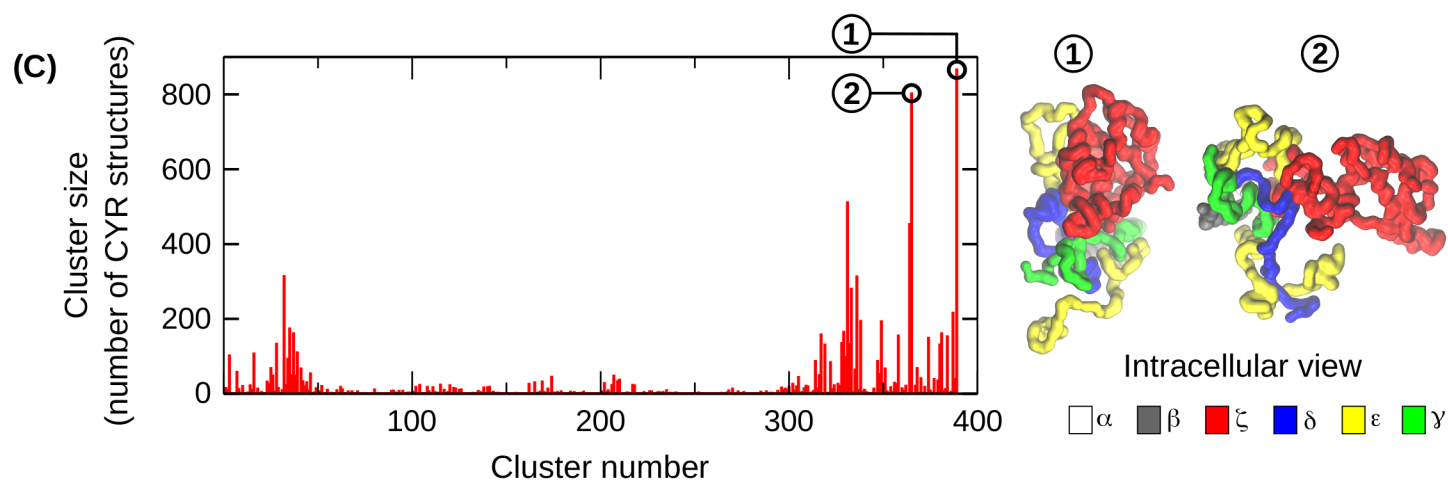

Supplement: S1 Fig — (A) Sequences of full-length subunits of the TCR-CD3 used for modelling. See Methods for Uniprot sequence IDs. Their secondary structure was predicted by the PSIPRED 4.0 server. The extracellular and intracellular residues modelled in this study are shown in boxes and underlined respectively. (B) Snapshot from one of the CGMD and ATMD simulations at simulation time (t) = 100 ns, starting from the same initial model (shown on the left). (C) TCR-CD3 cytoplasmic conformations grouped into clusters using a 3.5 Å RMSD cut-off, related to Fig 2A. The clusters containing the highest number of structures indicate the most stable conformation of the TCR-CD3 cytoplasmic region in our simulations. In (B) and (C), the structure is coloured by subunit: ζ:red, δ:blue, ε:yellow, γ:green, α:silver, β:black. (PDF) [file pcbi.1009232.s001.pdf]

(A)

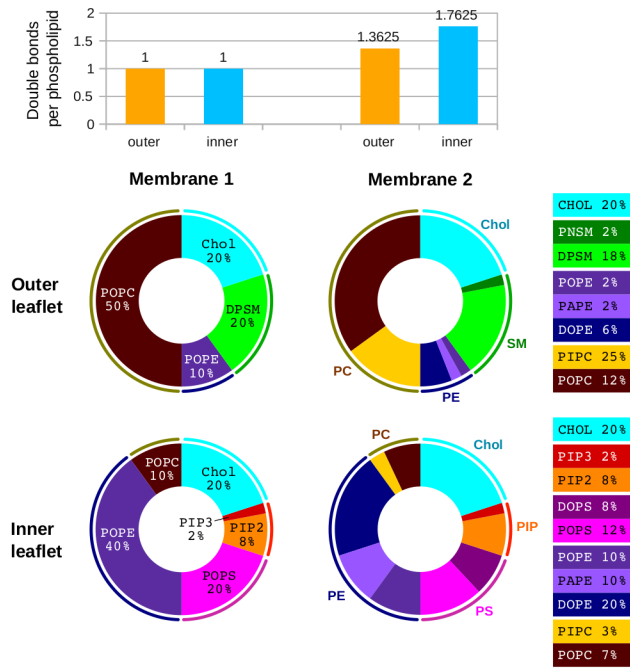

(B)

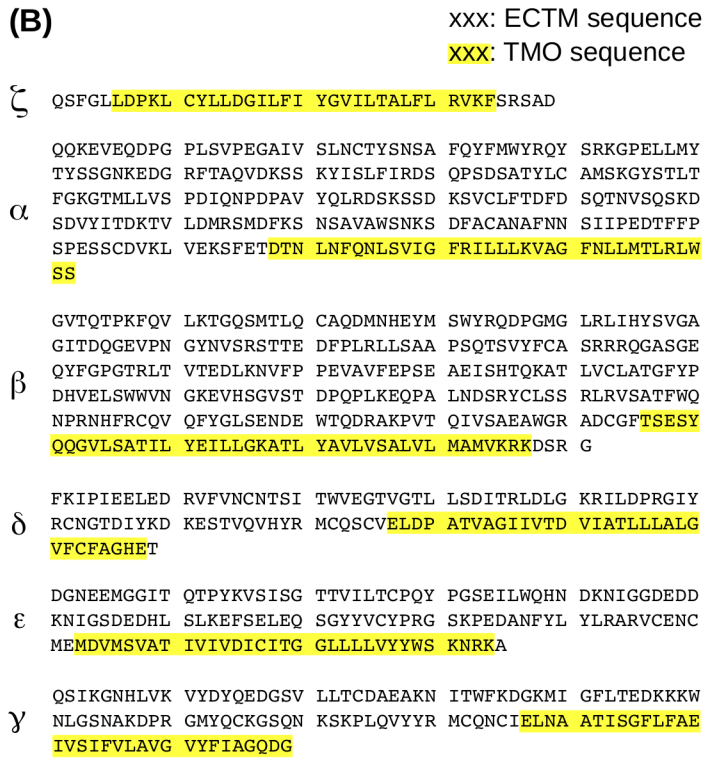

(C)

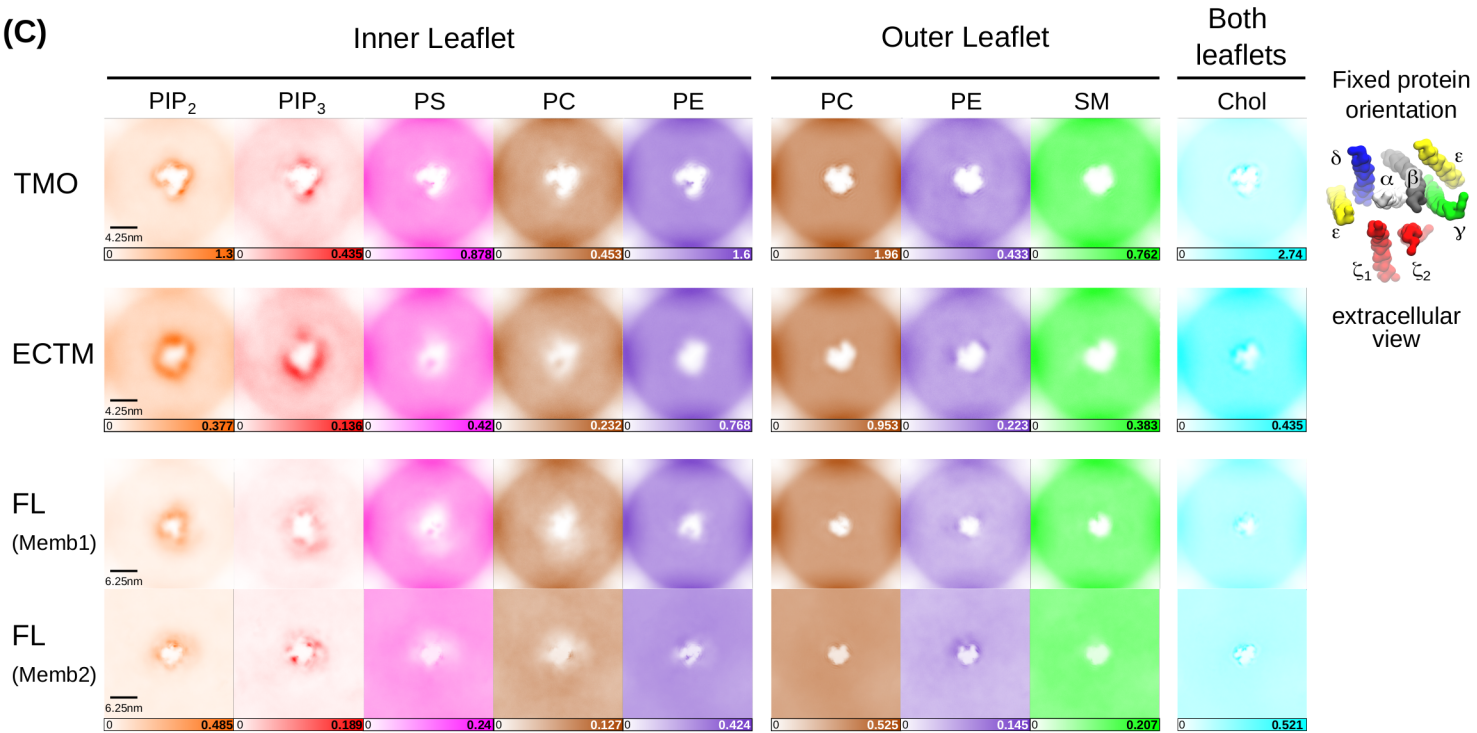

(D)

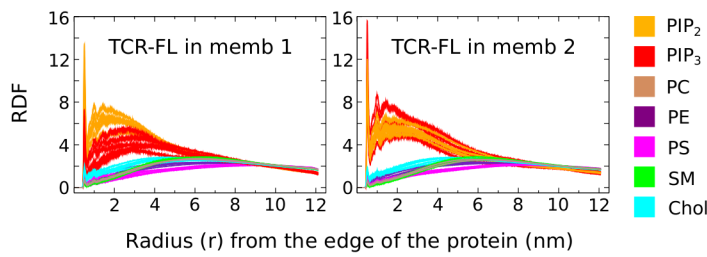

(E)

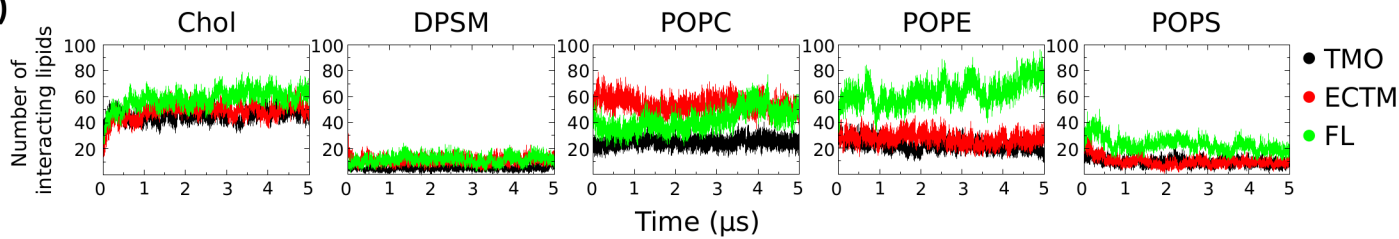

Supplement: S2 Fig — (A) Unsaturation levels of each leaflet of membranes 1 and 2 (top) and their lipid compositions (below), see also Tables 1 and 3. (B) Sequences of the ECTM (ectodomain and transmembrane), and TMO (transmembrane only) systems used for the CGMD simulations. The TMO sequences are a subset of the ECTM sequences, related to Fig 5A. (C) Extracellular view of the density of each lipid type in the membrane when the orientation of the TCR-CD3 is fixed in the center, related to Fig 5B. The colour gradient scale for each density displays the number of lipids corresponding to the minimum and maximum value. (D) Radial distribution functions of all lipid types in membrane 1 and 2. The different lines for each lipid type represent the RDF for the five repeat simulations that we performed for each system. (E) The average number of cholesterol, DPSM, POPC, POPE, and POPS lipids interacting with the TCR-CD3 over 5 μs time from all CGMD simulations of TMO, ECTM, FL systems conducted in membrane 1, related to Fig 5C. (PDF) [file pcbi.1009232.s002.pdf]

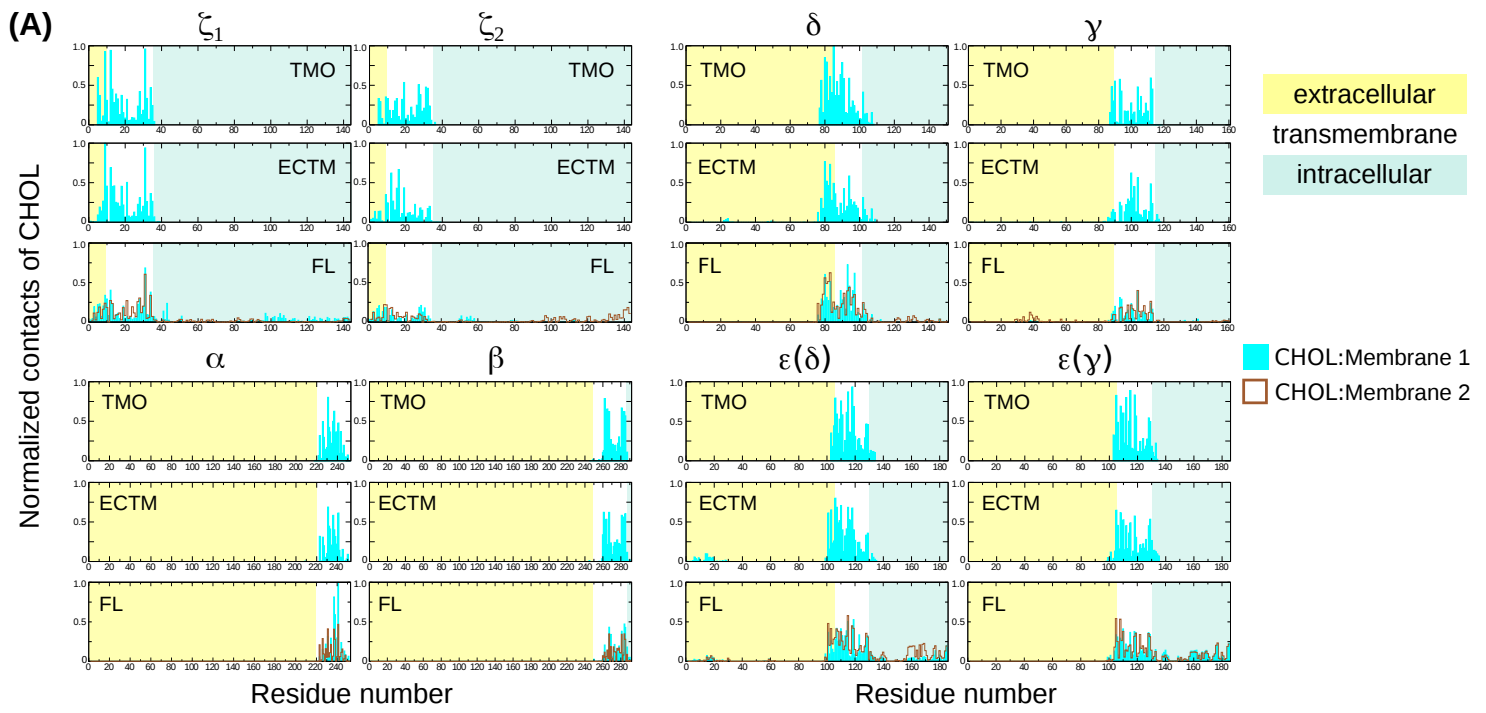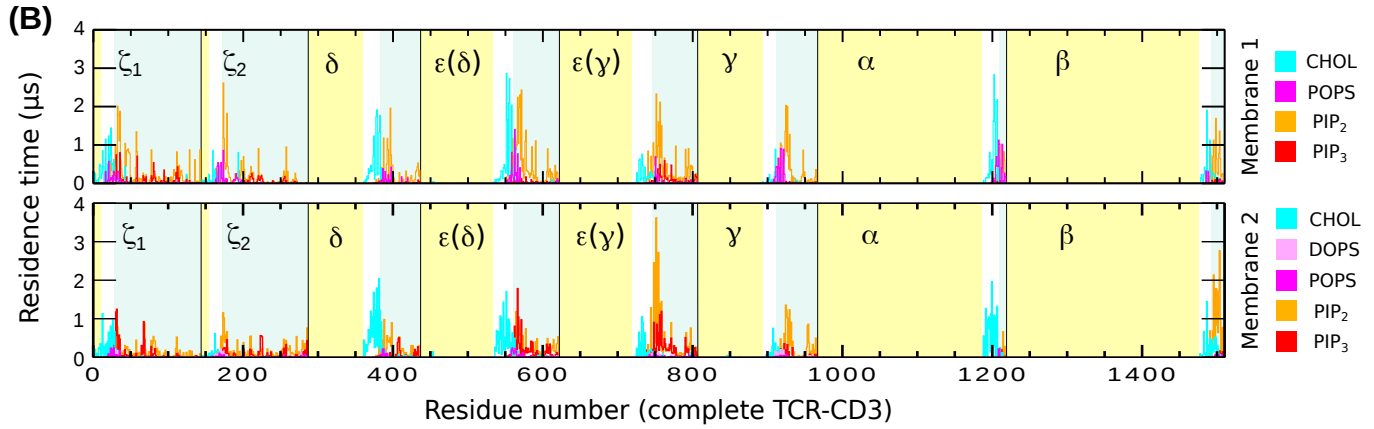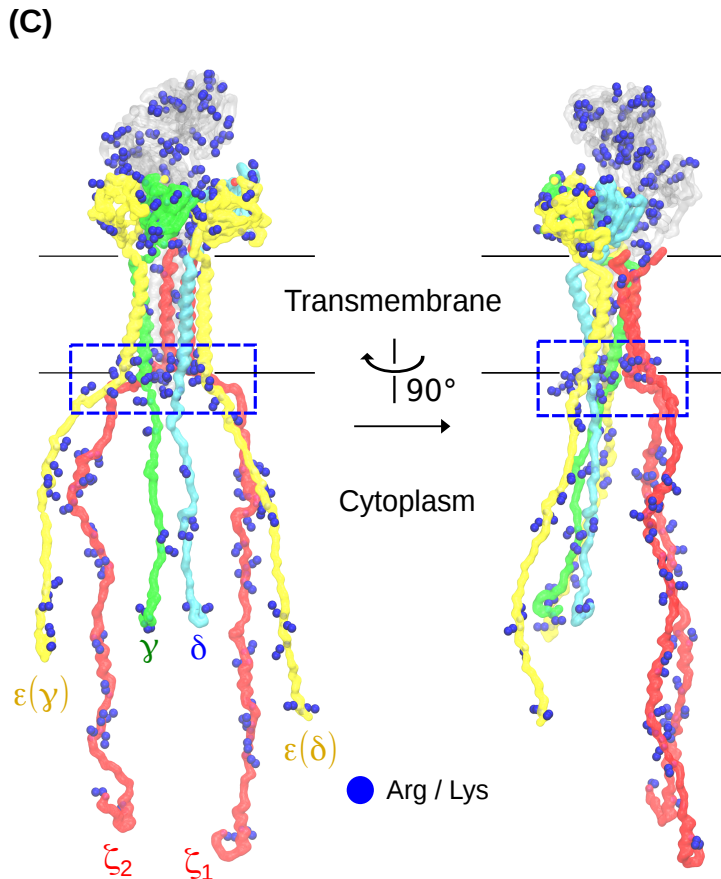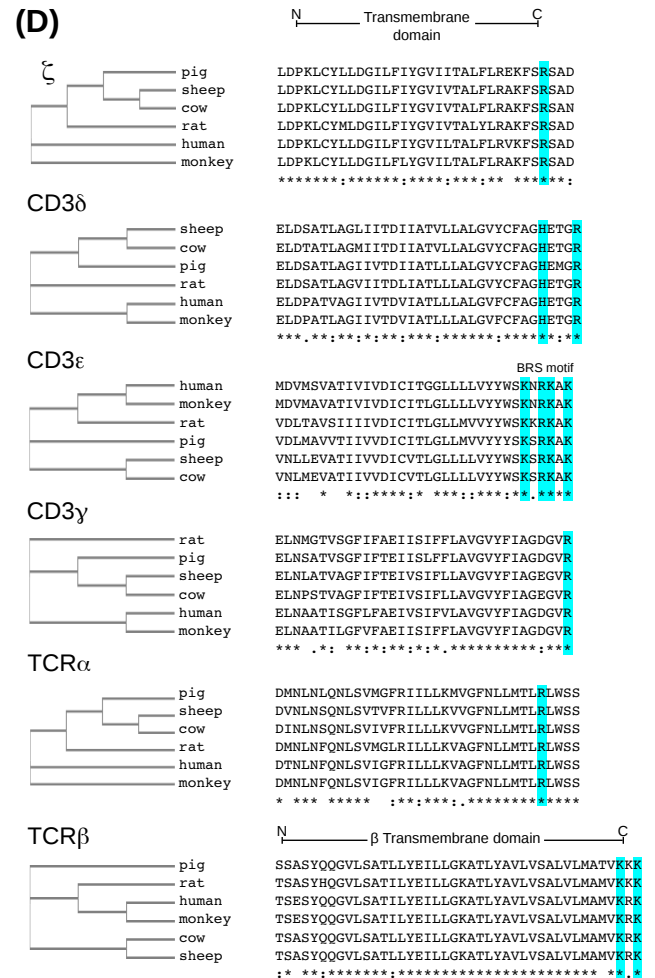

Supplement: S3 Fig — (A) Comparison of cholesterol interactions with the complete TCR-CD3 (FL), ECTM and TMO simulations. Normalization was done by dividing the contacts of each residue in the TCR-CD3 by the highest number of contacts. Therefore, the value 1 represents the highest contact while 0 represents no contact. The three TCR-CD3 systems: TMO, ECTM, FL, are normalized separately. (B) Residence times of cholesterol and anionic lipids shown for membrane 1 and 2. (C) The cationic anchor of the TCR-CD3, related to Fig 5E, located at the interface of the TMR and CYR is indicated within a box. (D) Multiple sequence alignment of the TMR and juxtamembrane residues of all TCR-CD3 subunits, related to Fig 5E, indicates the conservation of cationic residues at the TMR-CYR interface across different species i.e. Homo sapiens (humans), Rattus norvegicus (rat), Sus Scrofa (pig), Bos taurus (cow), Ovis aries (sheep), and Macaca mulatta (monkey). (PDF) [file pcbi.1009232.s003.pdf]

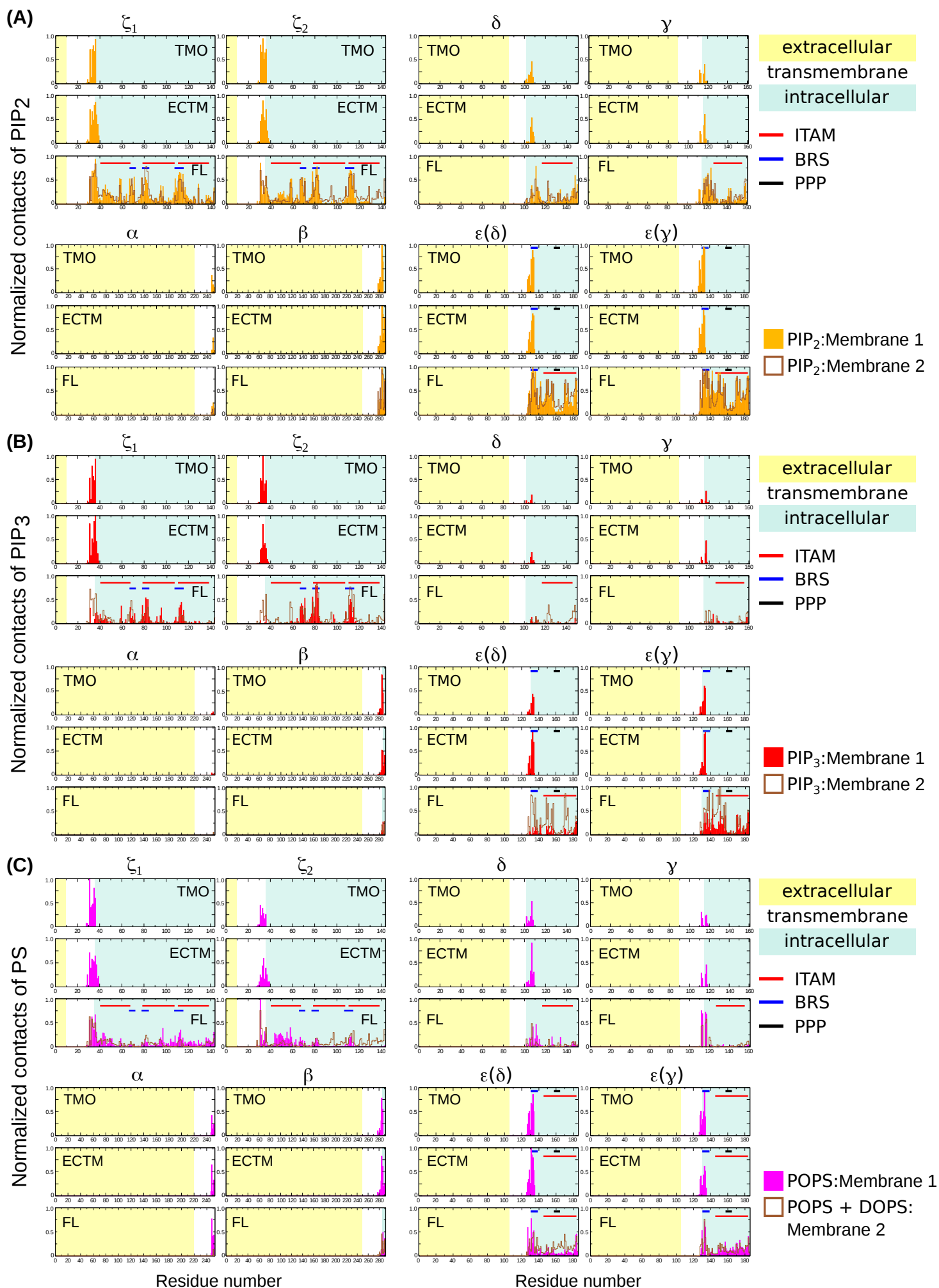

Supplement: S4 Fig — Interactions of (A) PIP2, (B) PIP3, and (C) PS with the complete TCR-CD3 (FL), ECTM and TMO simulations. Normalization is done by dividing the contacts of each residue in the TCR-CD3 with a lipid type by the highest number of contacts with that lipid type. Therefore, the value 1 represents the highest contact while 0 represents no contact. The three TCR-CD3 systems: TMO, ECTM, FL, are normalized separately. (PDF) [file pcbi.1009232.s004.pdf]

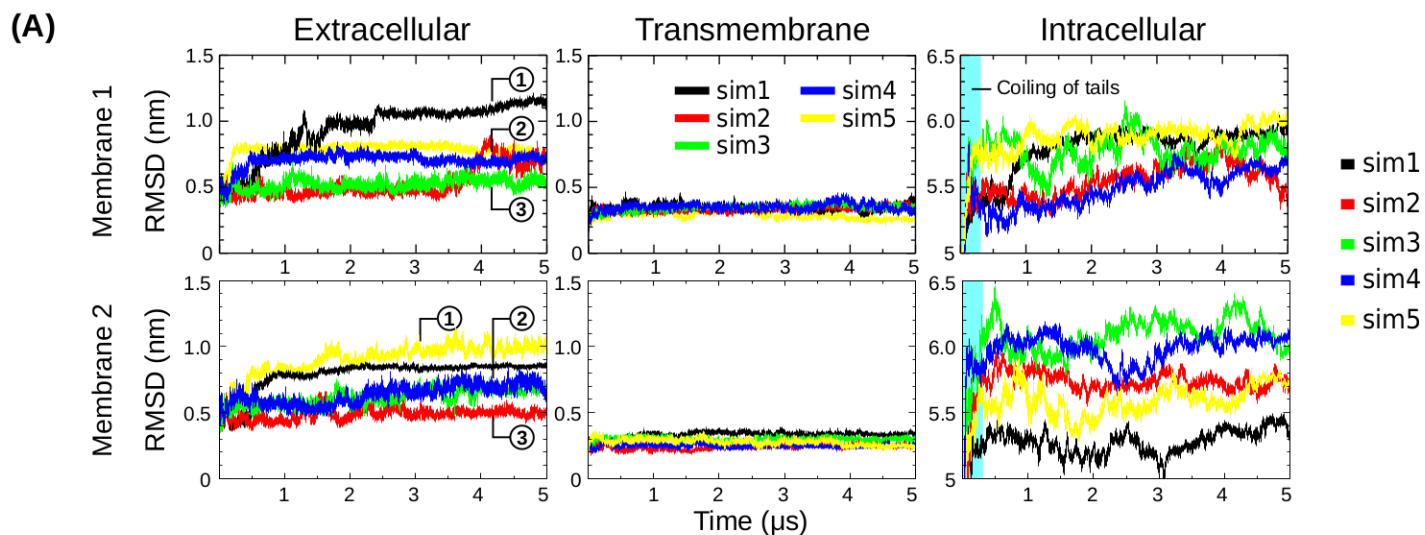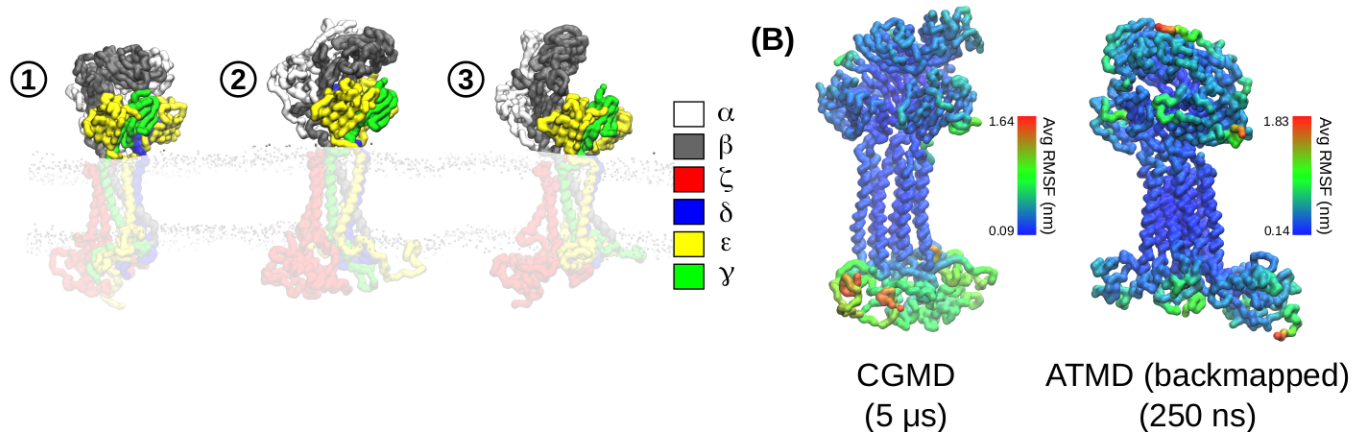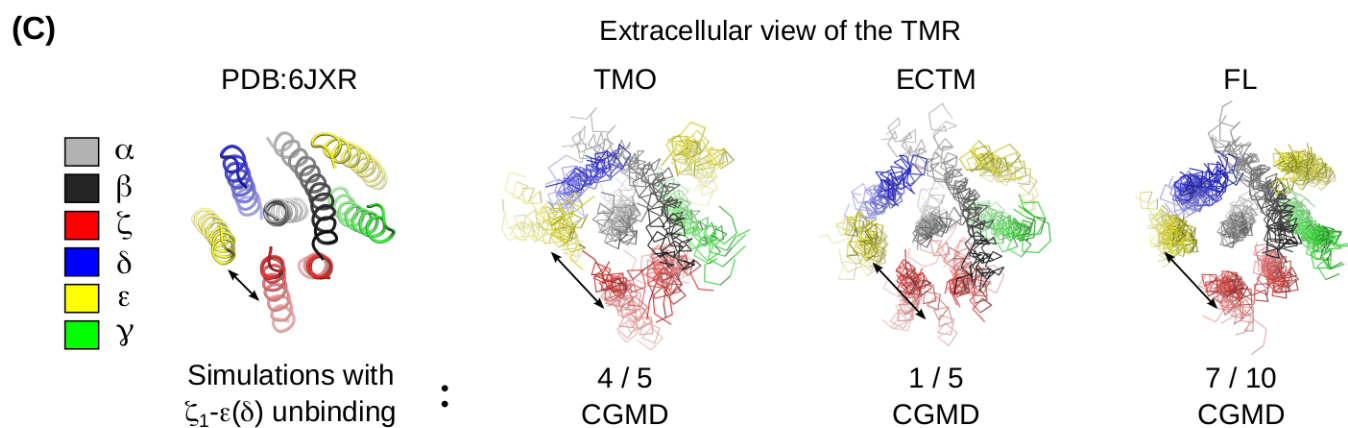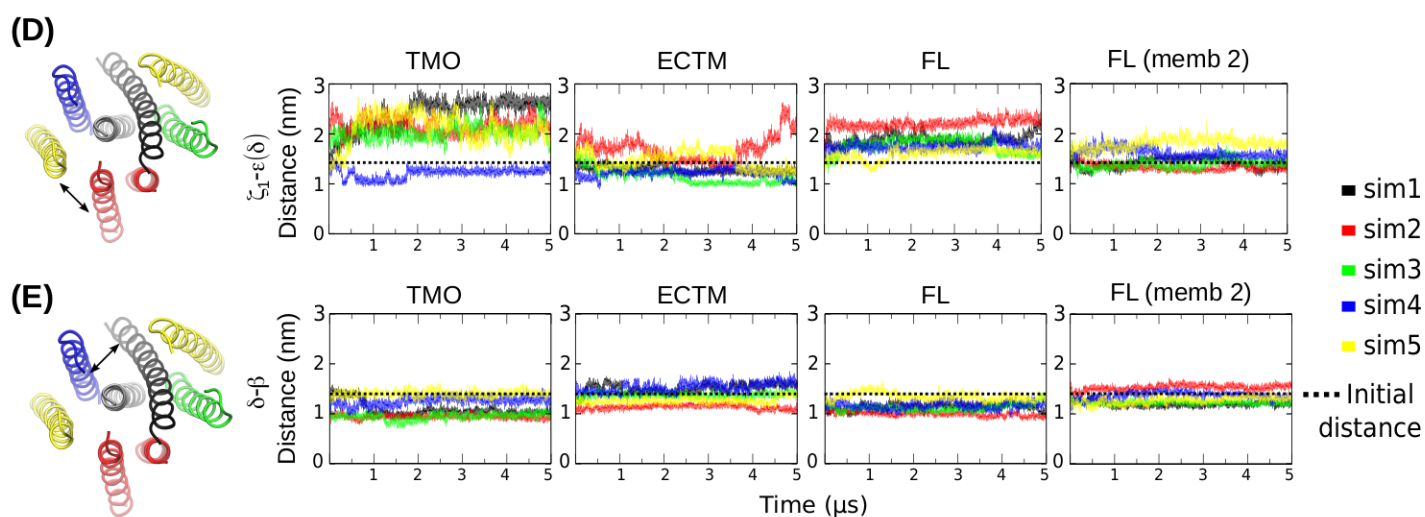

Supplement: S5 Fig — (A) Backbone RMSD of the TCR-CD3 ECD, TMR, and CYR in membrane 1 and 2. (B) Backbone RMSF of the complete TCR-CD3 in the CGMD and ATMD simulations. A structure extracted at 1 μs was used a reference for this RMSF calculation. (C) Extracellular view of five TMR snapshots aligned from the end of the TMO, ECTM, FL simulations indicating the frequency of ζ1-ε(δ) dissociation compared to the cryo-EM TMR structure (PDB:6JXR). (D) Distance between the center of mass of ζ1 and of ε(δ) subunits, and (E) distance between the center of mass of δ and of β subunits in all simulations systems over 5 μs compared to their initial distances calculated from the cryo-EM structure. (PDF) [file pcbi.1009232.s005.pdf]

(A) TMR

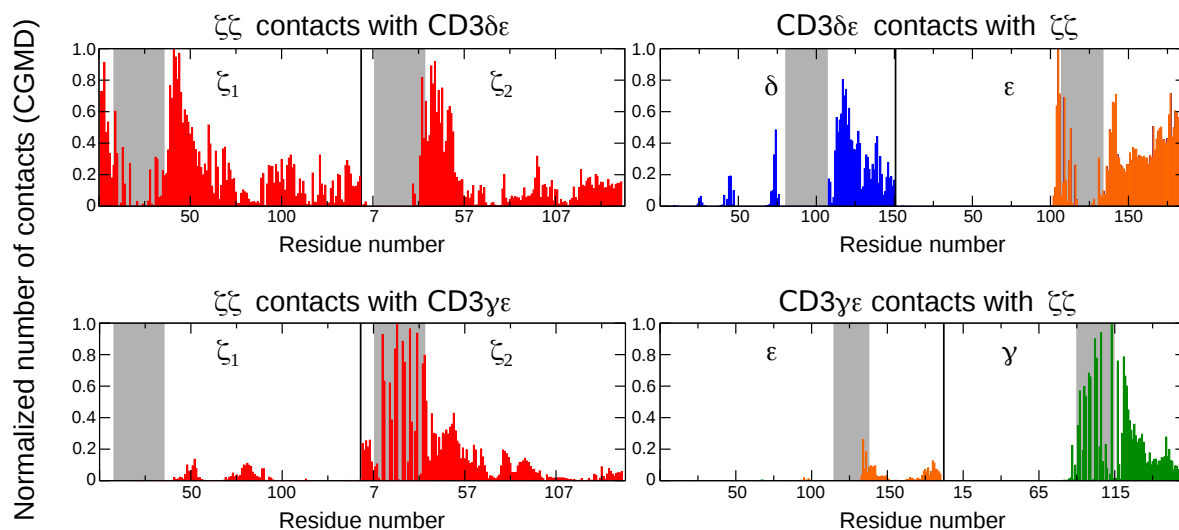

(B) V $\alpha$ V $\beta$  TMR

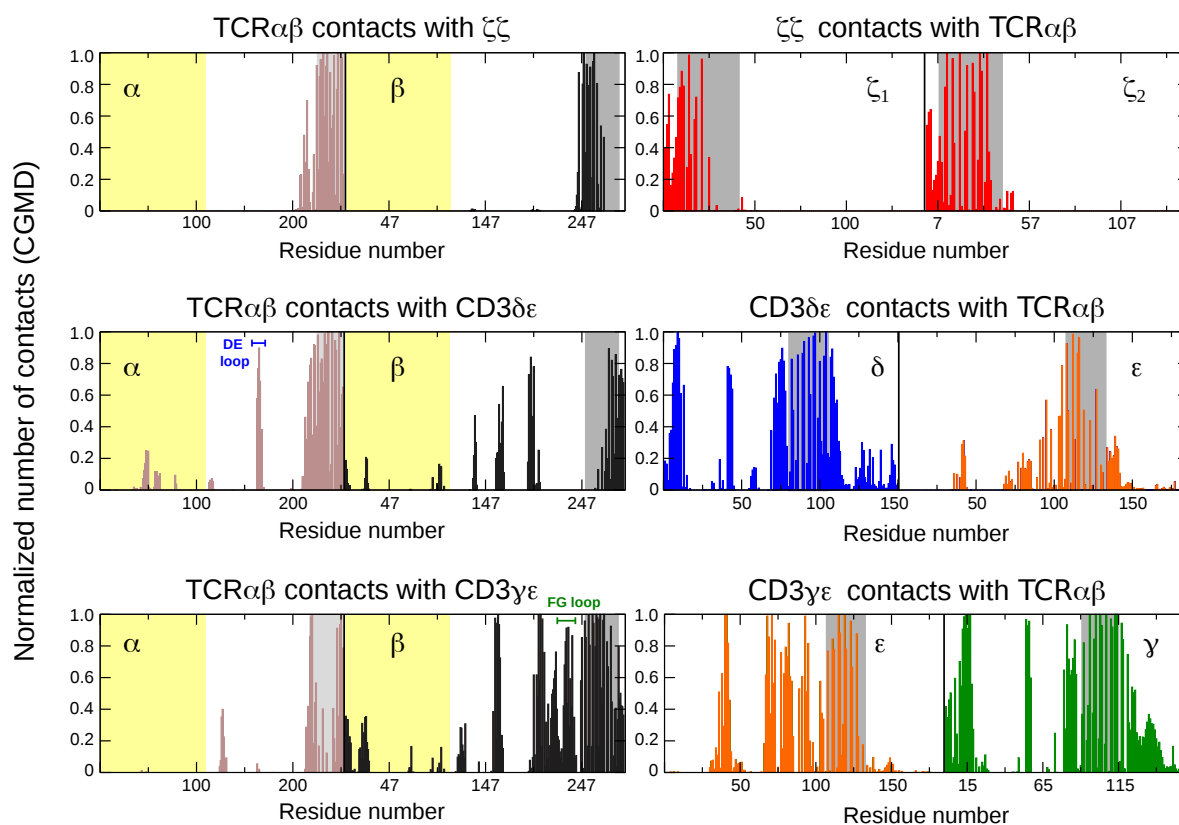

(C)

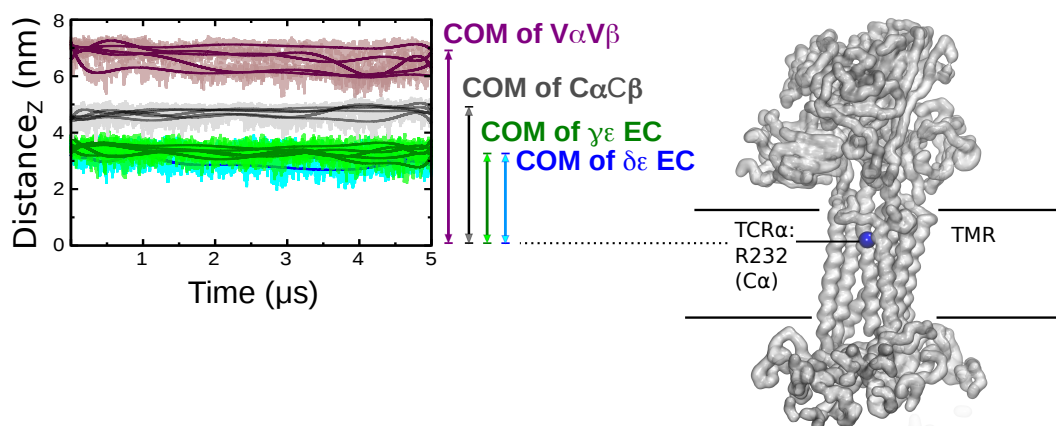

Supplement: S6 Fig — Normalized full-length inter-chain interactions of (A) the ζζ dimer with the CD3δε and CD3γε dimers, and (B) the TCRαβ dimer with the ζζ, CD3δε, and CD3γε dimers in CGMD simulations. Normalization is done by dividing the contacts of each residue by the highest number of contacts within each dimer. (C) Distance of the center of mass of the TCRαβ variable domain (VαVβ), the TCRαβ constant domain (CαCβ), the CD3δε, and CD3γε ectodomains (EC), each to the Cα atom of TCRα:R232 residue in the TMR along the vertical (Z) axis. The smoothened lines are a polynomial regression to the 10th degree of the distances versus time. (PDF) [file pcbi.1009232.s006.pdf]

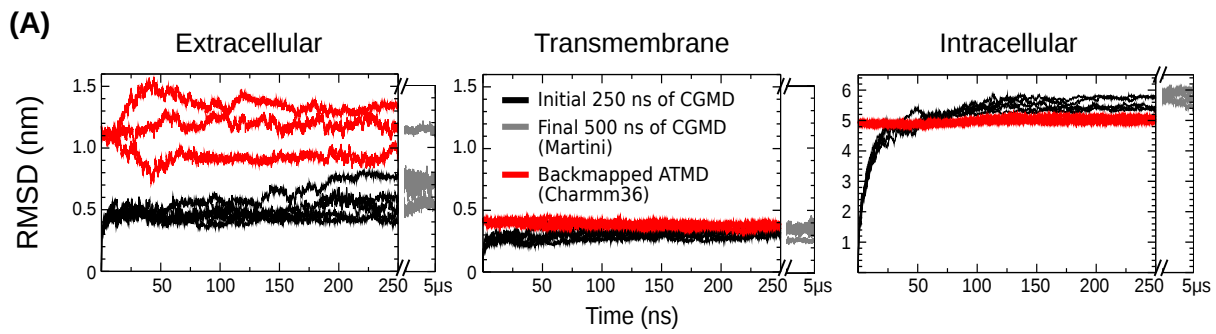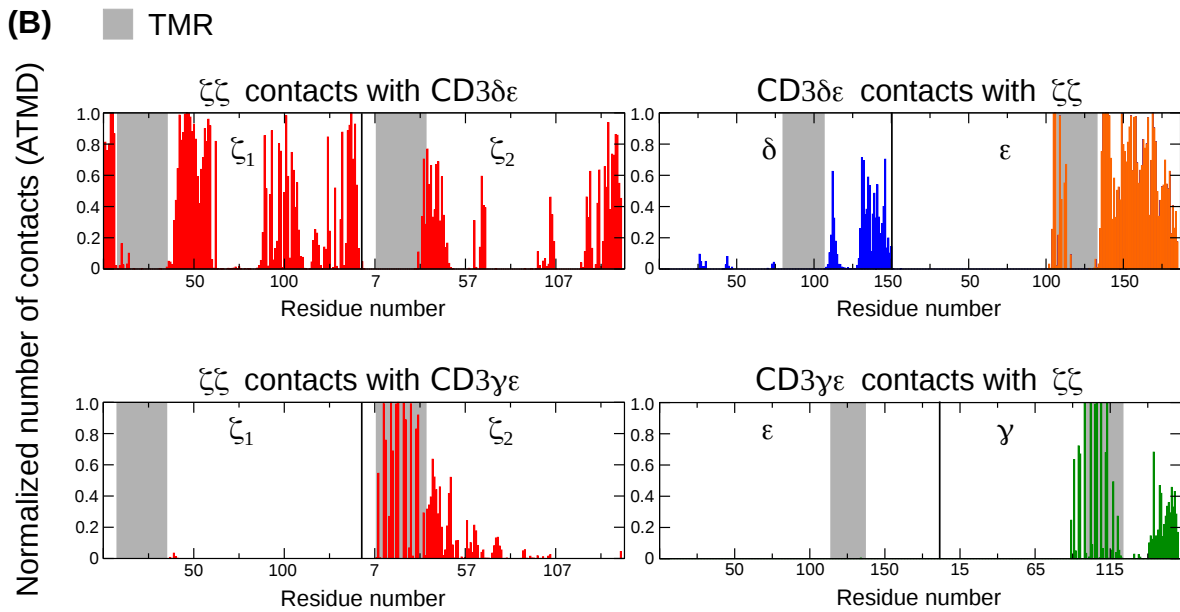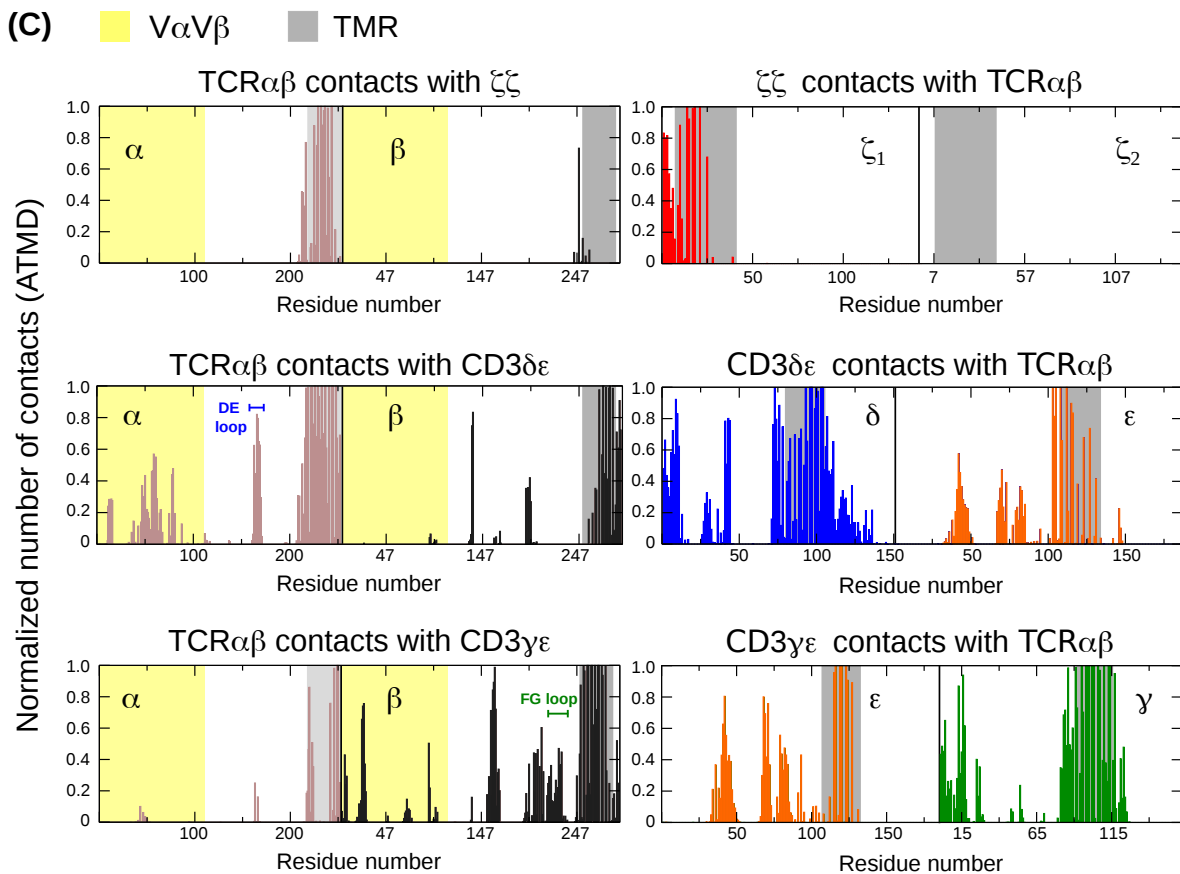

Supplement: S7 Fig — (A) Backbone RMSD of the ECD, TMR, and CYR of the TCR-CD3 in the initial 250 ns of the CGMD and the backmapped ATMD simulations. Backbone RMSDs during the last 500 ns of CGMD are also shown for comparison. The initial model of the TCR-CD3, as seen in Fig 1, was used as a reference for this calculation. (B) Normalized full-length inter chain interactions of the ζζ dimer with the CD3δε and CD3γε dimers, and (C) the TCRαβ dimer with the ζζ, CD3δε, and CD3γε dimers in ATMD simulations. Normalization is done by dividing the contacts of each residue by the highest number of contacts within each dimer. (PDF) [file pcbi.1009232.s007.pdf]

Number of  $\alpha$ -helix forming residues in the cytoplasmic region (ATMD)

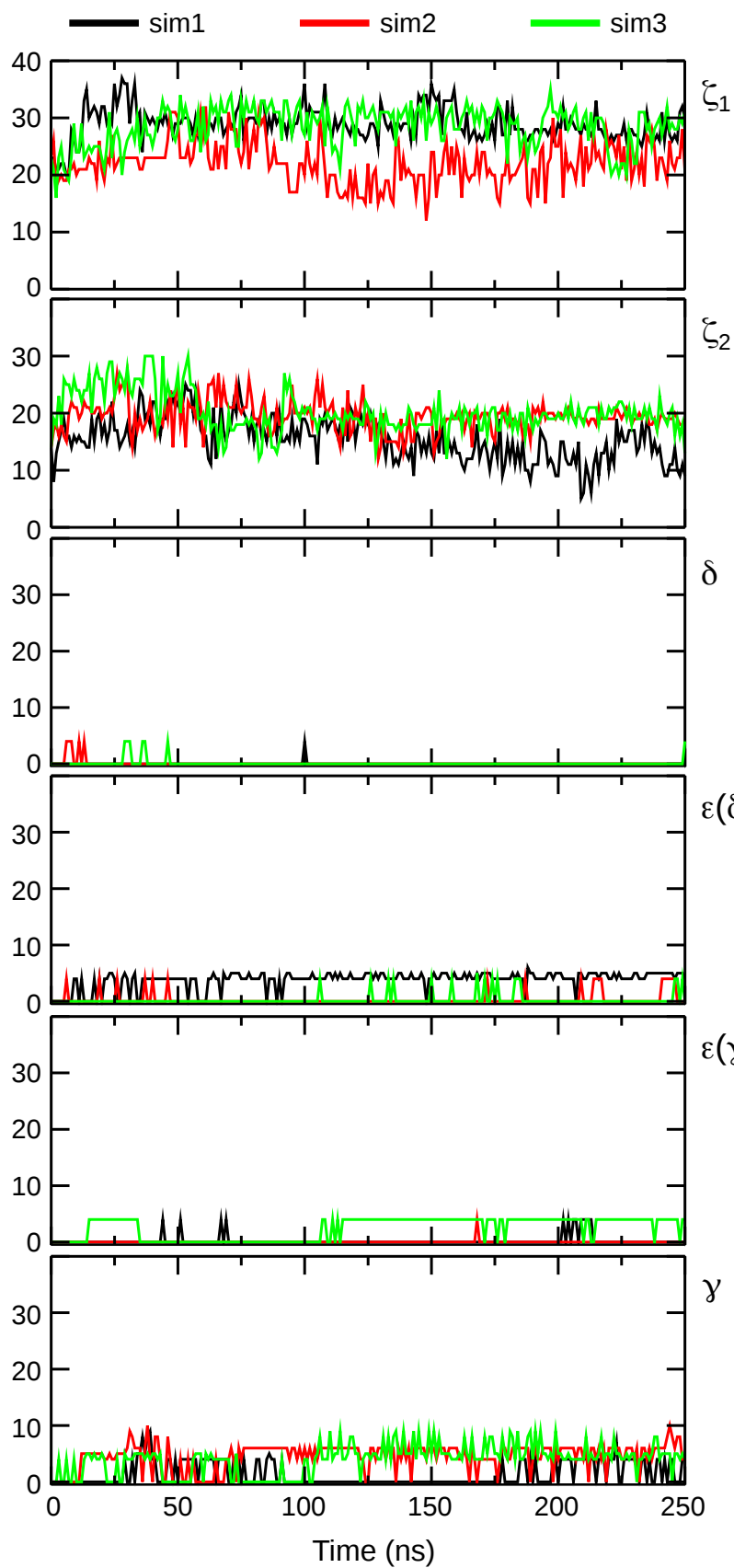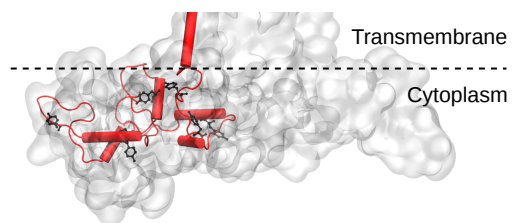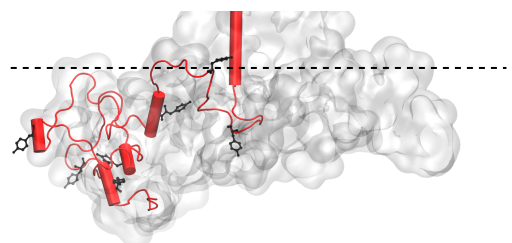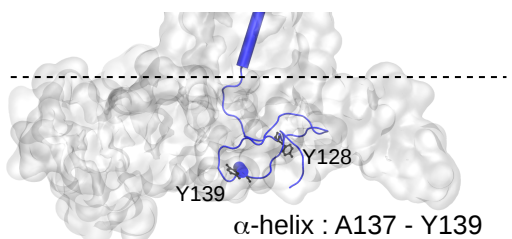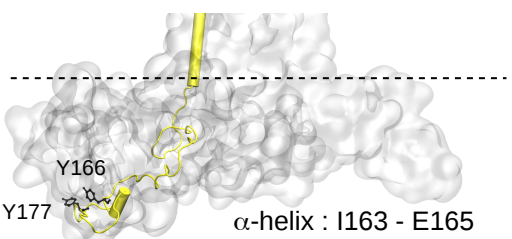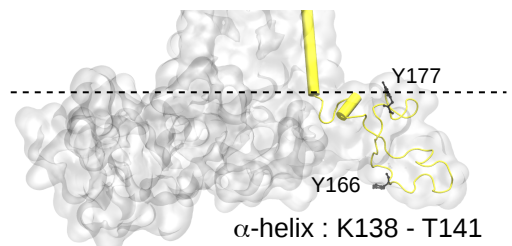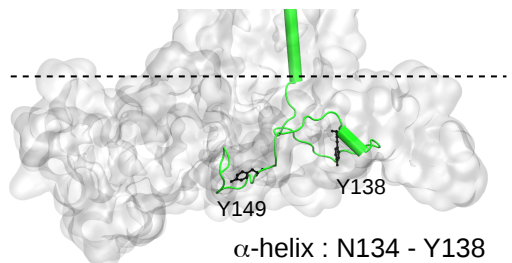

Supplement: S8 Fig — Number of α-helix forming residues in the CD3 and ζ CYRs versus time from all three backmapped ATMD simulations that were run for 250 ns (left). Location of cytoplasmic α-helices relative to the ITAM tyrosines of the respective subunits (right). The ITAM tyrosines and α-helix forming residues in the CD3 subunits observed at the end of 250 ns in simulation-3 are labelled. The entire protein is shown using the surface representation, the ITAM tyrosines are represented as black ball and sticks, and the subunits are shown in cartoon representation and coloured as follows: ζ:red, δ:blue, ε:yellow, γ:green (right). (PDF) [file pcbi.1009232.s008.pdf]
